# Chondrosarcoma: Adjuvant Therapeutic Effects of the ASPH Small Molecule Inhibitor, SMI-1182, With Doxorubicin

**Published:** 2025-11-15

**Authors:** Keisuke Oyama, Megan Fife, Rolf I. Carlson, Ming Tong, Yoshihiro Matsumoto, Yuki Yokota, Bhaskar Das, Parthiban Chokkalingam, Rene Rodriguez, Suzanne M de la Monte

**Affiliations:** 1 Department of Orthopedic Surgery and Gastroenterological Surgery, Rhode Island Hospital, Alpert Medical School at Brown University, USA and Graduate School of Medicine, Osaka University, Osaka, Japan; 2 Molecular Pharmacology, Physiology, and Biotechnology Graduate Program at Brown University, USA; 3 Department of Medicine, Rhode Island Hospital, Alpert Medical School at Brown University, USA; 4 Drug and Biotherapeutic Discovery University at Buffalo, The State University of New York (SUNY), Buffalo, USA; 5 Health Research Institute of Asturias (ISPA), University Institute of Oncology of Asturias (IUOPA), Oviedo, Spain, and CIBER en oncologia (CIBERONC), Madrid, Spain; 6 Departments of Pathology and Laboratory Medicine, Neurology, and Neurosurgery, Alpert Medical School at Brown University, USA

**Keywords:** Aspartyl-asparaginyl-β-hydroxylase, Cancer treatment, Chondrosarcoma, Doxorubicin, Notch, Small molecule inhibitor

## Abstract

Chondrosarcoma (CS) is the most common adult malignant bone tumor. Its poor prognosis is due to chemotherapy resistance and high rates of post-treatment recurrence. The finding that CSs express abundant Aspartyl-Asparaginyl-Β-Hydroxylase (ASPH), which drives invasive tumor growth via Notch pathway activation, provides a new opportunity for treatment in combination with standard Doxorubicin (DOX) chemotherapy. We hypothesized that the small-molecule inhibitor SMI-1182, which targets the catalytic domain of ASPH, could enhance the chemotherapeutic effects of DOX by further reducing CS cell viability, motility, and invasive growth. Human CS-1 and CDS11 conventional chondrosarcoma cell lines were treated with broad dose ranges of DOX, SMI-1182, or DOX+SMI-1182 to measure their separate and combined effects on cell viability, toxicity, motility, and invasive growth. Mechanistic studies included assessments of genes and proteins pertinent to ASPH expression and Notch signaling. Treatment with DOX or SMI-1182 caused dose-dependent CS-1 and CDS11 cell loss and cytotoxicity. SMI-1182, with or without DOX, significantly reduced directional motility and invasive growth relative to vehicle. Combined treatments with DOX and SM-1182 had synergistic cytotoxic and anti-invasive growth effects linked to reduced expression of ASPH immunoreactivity, Notch transcription factors, and insulin receptor substrate, type I, which positively regulates both ASPH and Notch. Dual treatment with DOX and SMI-1182 could potentially improve disease-free survival with CS by simultaneous targeting of multiple upstream mediators of aggressive malignant tumor behavior.

## Introduction

Chondrosarcoma (CS) is the most common bone sarcoma in adults [1] and accounts for 20%–25% of osseous malignancies. The initial diagnosis of CS is typically made in people 30 to 60 years old, but 80% are older than 40. Among the four main histopathological subtypes, conventional CS accounts for 75%, and dedifferentiated, mesenchymal and clear cell subtypes together account for approximately 25% of CS. In the U.S.A., the crude annual incidence of CS reportedly increased over time through 2013 [2], but subsequently the rates leveled off. In a later comprehensive review, Thorkildsen and Myklebust showed that the prevalence rate of CS progressively increased between 2000 and 2020, although age-adjusted incidence and mortality did not fluctuate significantly over that same interval [3]. Higher prevalence vis-à-vis stable incidence rates may suggest improvements in survival or detection of treatable recurrent disease.

The standard treatment for CS is wide surgical resection, including the affected bone and surrounding tissue with wide negative margins [4]. In patients with unresectable chondrosarcoma or metastatic disease, the clinical prognosis is poor [4]. The first-line chemotherapeutic agent for high-grade and advanced sarcomas, including chondrosarcoma, consists of Doxorubicin (DOX) [5,6]. However, high-grade conventional chondrosarcomas are known to be resistant to radiotherapy and chemotherapy [4], and DOX monochemotherapy is suboptimum in terms of efficacy, as evidenced by the universally poor outcomes due to pulmonary metastases and mortality [7]. Current standard treatment regimens mainly consist of multimodal combination treatment with 2 or 3 drugs including doxorubicin plus DNA damaging agents such as cisplatin or isofamide. However, unresectable recurrences and metastasis linked to post-treatment resistance to anticancer agents render chondrosarcoma clinical outcomes discouragingly poor [4,8].

The five-year survival rate with CS is between 26% and 32%, with metastatic disease further reducing mean survival to 23% [9]. Importantly, prognosis is linked to tumor grade such that high-grade CSs exhibit the uppermost recurrence rates and shortest survivals [9]. Moreover, there is no effective systemic treatment for chondrosarcoma. These statistics combined with the minimal progress in CS therapeutics led the American Academy of Orthopaedic Surgeons to prioritize the development of CS treatment strategies. Most likely, newer more effective therapeutics will require molecular targeting to inhibit CS growth and metastasis. Potential strategies to improve survival with CS include the use of chemotherapeutic agents that target signaling pathways that have roles in chondrosarcoma malignant behavior such as PI3K-Akt-mTOR pathway and angiogenesis [10,11], in addition to genomic mutations of Isocitrate Dehydrogenase (IDH) IDH1/2, exostosin-1/2 (EXT1/2), COL2A1, TP53, Telomerase Reverse Transcriptase (TERT), and cyclin-dependent kinase inhibitor 2A/B (CDKN2A/B) [4,10]. To this end, there are many on-going but early-stage clinical trials designed to assess the therapeutic efficacy in targeting angiogenesis (PD-L1), mTOR, PI3K, CDK4/6, or IDH1 [4]. In a retrospective study, Molho, et al. [12] demonstrated efficacy in treating CS with the mTOR inhibitor sirolimus in combination with cyclophosphamide [8].

Another likely important therapeutic target for chondrosarcoma is Notch which regulates cell proliferation, differentiation, migration, infiltrative growth, and cell fate, and has potential therapeutic efficacy for a broad range of cancers [13–17]. Aberrantly increased Notch activity has been implicated in many malignancies, including chondrosarcoma [18]. Notch signaling inhibitor, LY3039478 was initially reported to be well tolerated as a monotherapy for various carcinomas [19], but in a later Phase 1b/II randomized study, although intra-tumoral Notch and downstream signaling through phosphorylated Akt were reduced, there were no significant effects on progression-free or overall survival in a heterogenous group of sarcomas, including CS [20]. Furthermore, enthusiasm for pursuing Notch-inhibitor chemotherapeutic drugs for advanced disease and advancing their use in clinical trials has been dampened by the significant off-target effects [16,21–23].

Emerging data suggest that Aspartyl-Asparaginyl-Β-Hydroxylase (ASPH) is an excellent candidate for strategically targeting Notch signaling networks in malignancies. ASPH, a type II transmembrane protein of the α-ketoglutarate-dependent dioxygenase family [24], has functional roles in cell motility utilized for infiltrative and metastatic tumor growth [25]. Mechanistically, the C-terminal region of ASPH contains a catalytic site that hydroxylates Epidermal Growth Factor (EGF)-like domains expressed in molecules such as Notch and Jagged [26–29]. EGF-like domains have important roles in regulating localized signaling of growth and differentiation [30]. ASPH’s interactions with Notch lead to nuclear translocation of the Notch intracellular domain [31] followed by the activation of transcription factors such as HES and HEY [27,31]. Abolishment of ASPH’s catalytic activity by either site-directed mutagenesis of its critical His-675 residue or deletion of the molecule’s C-terminus, prevents Notch activation, and reduces cell motility and tumor growth [26].

Previous studies demonstrated high levels of ASPH expression in clinically aggressive malignancies, such as those originating from the gastrointestinal tract, lung, liver, pancreas, breast, or central nervous system [27,31–35]. Furthermore, high levels of ASPH expression were shown to correlate with worse clinical outcomes [27,36] including abbreviated survival [34,37]. In contrast, most normal cells and tissues express very low or non-detectable levels of ASPH [32]. Similarly, preliminary studies also suggest that high-grade conventional chondrosarcomas express higher levels of ASPH than low-grade chondrosarcomas [38]. In light of the well-documented roles of Notch in many malignancies including chondrosarcoma, ASPH could potentially serve as an excellent target for chondrosarcoma diagnostics and therapeutics [39–42].

One evolving approach for cancer therapeutics is the use of small molecule inhibitors that target critical signaling pathways utilized in the growth and survival of malignant cells including inhibitors of tyrosine and serine/threonine kinases, proteosomes, apoptosis, or matrix metalloproteinases [43]. Of note is that a small molecule inhibitor of ADAM (INCB7839) designed to release the extracellular domain of Notch, was shown to be an effective anti-cancer agent in epithelial neoplasms [44,45]. Regarding ASPH, previous studies led to the design and characterization of unique small molecule inhibitors that fit in the pocket of ASPH’s catalytic domain and diminish or abolish its enzymatic (hydroxylase) activity [25,33,34,36,46,47]. The ASPH small molecule inhibitor 1182 (SMI-1182) was shown to inhibit ASPH’s functions linked to Notch activation [34,46,47] and produce anti-tumor effects in experimental models of cholangiocarcinoma [46], hepatocellular carcinoma [36], pancreatic carcinoma [33], breast cancer [25], glioblastoma [34], and more recently, chondrosarcoma [38]. However, despite encouraging results, none of the studies demonstrated complete cancer cell killing with SMI-1182 used as a monotherapy. The preliminary finding that the combined use of SMI-1182 with Dox in an experimental breast cancer model was more effective than SMI-1182 monotherapy [48] inspired further investigation of this concept in relation to chondrosarcoma.

We hypothesized that SMI-1182 could be used as an adjuvant chemotherapeutic agent to enhance DOX-mediated CS killing. This concept has clinical relevance because if SMI-1182 synergistically increases the chemotherapeutic effects of Dox, then more effective CS treatment could be provided at lower, less toxic doses of Dox. Herein, we report results obtained with in vitro experimental models to assess the potential for future in vivo therapeutics to improve clinical outcomes in patients with CS.

## Materials and Methods

### Cell Culture and treatments

The studies utilized two mycoplasma-free human CS cell lines: CS-1 (RRID: CVCL_T022) and CDS11 (RRID: CVCL_WJ30). The CS-1 cell line originated from a high-grade (Grade 3) conventional CS tumor in an untreated patient with metastatic disease [49]. CDS11 cells correspond to slow growing, less invasive secondary conventional CS [50,51]. The CS-1 and CDS11 cells were authenticated using short tandem repeat profiling, and all studies were performed with cells passaged 10 or fewer times. CS-1 cells were maintained in Roswell Park Memorial Institute (RPMI) 1640 medium (Cytiva/Hyclone, Logan, UT, USA) and CDS11 cells were grown in Dulbecco’s Modified Eagle’s Medium (DMEM) with high glucose (Cytiva/Hyclone, Logan, UT, USA). Culture media for both CS-1 and CDS11 was supplemented with 5% Fetal Bovine Serum (FBS) and 2mM L-Glutamine (Corning, Glendale, AZ, USA), and all in vitro experiments were conducted at 37°C in a standard humidified 5% CO_2_ incubator. To study the effects of DOX, SMI-1182, and DOX+SMI-1182, 24-hour-old sub-confluent CS cultures were treated vehicle (DMSO; negative control), 0–10 μM DOX (Pfizer Inc., New York, NY, USA), 0–100 μM SMI-1182, or varied concentrations of DOX+SMI-1182 by direct addition to the medium. After 48h of treatment, the cells were analyzed for viability, cytotoxicity, morphology, ASPH immunoreactivity, directional motility, colony formation, invasion, and gene expression related to Notch pathway and insulin, Insulin-Like Growth Factor (IGF), and Insulin Receptor Substrate (IRS) networks. The insulin/IGF/IRS signaling pathways were of interest because of their regulatory roles in relation to ASPH and Notch [27,52,53].

### Cell viability and cytotoxicity assays

Cell viability and metabolic function were measured with the 3-(4,5-dimethylthiazol-2-yl)-2,5-diphenyltetrazolium bromide (MTT) (#M5655, Sigma-Aldrich, St. Louis, MO, USA) assay [54–56]. Cell density was assessed by Hoechst 33342 (H3570, Invitrogen, Carlsbad, CA, USA) fluorescence. Cytotoxicity was measured with the CyQUANT Cytotoxicity Assay Kit (G6PD Release) (Thermo Fisher Scientific, Bedford, MA USA) which is based on glucose-6-phosphate dehydrogenase release into the culture supernatant. All three assays were performed in 96-well cultures with 3000 viable (Trypan blue-excluded) CS cells initially seeded in 100μL of culture medium. The MTT assay was performed by adding 10μL of freshly prepared MTT solution (5 mg/mL in MEM without phenol red) to each well and incubating the plate for 20 minutes at 37°C in a 5% CO_2_ atmosphere. After aspirating the culture medium, the substrate was eluted by the addition of 100μL of acidic isopropanol elution buffer (0.04 mol/L HCl/isopropanol) and 5 minutes of gentle platform agitation at room temperature. Absorbances were measured at 540 nm using a Spectra-Max M5 Multimode Plate Reader (Molecular Devices, Sunnyvale, CA, USA) [54–56]. To measure cell number in the same wells as MTT activity, the eluate buffer was replaced with 50μL of 10 μg/mL Hoechst 33342 dye in Phosphate Buffered Saline (PBS). Fluorescence intensity (Ex360 nm/Em460 nm) was measured in a Spectra-Max M5 after 5 minutes room temperature incubation with light-shielding. Cytotoxicity was quantified in 50μL of culture supernatant added to CyQUANT assay solution and incubated for 30 minutes. Fluorescence intensity (Ex530 nm/Em590 nm) was measured in a Spectra-Max M5.

### Sample Preparation for Immunoassays

CS cells seeded into 6-well plates (1 × 10^5^/mL) were treated for 48h with Vehicle (DMSO), DOX (0.05μM), low dose SMI-1182 (3.125μM), high dose SMI-1182 (25μM), low-dose SMI-1182 + DOX, or high dose SMI-1182 + DOX. For protein assays, the cells were harvested in weak lysis buffer (50 mM Tris (pH 7.5), 150 mM NaCl, 5 mM EDTA (pH 8.0), 50 mM NaF, 0.1% Triton X-100) containing protease inhibitor cocktail (1mM PMSF, 0.1 mM TPCK, 2 μg/ml aprotinin, 2 μg/ml pepstatin A, 1 μg/ml leupeptin). After centrifuging the homogenates at 14,000 rpm for 10 minutes at 4°C, the supernatant fractions were divided and stored at −80°C. Prior to freezing, small aliquots were set aside to measure protein concentration with the Bicinchoninic Acid (BCA) assay. Proteins harvested from 6-well cultures and treated as described above were used for Western blot analysis and Enzyme-Linked Immunosorbent Assays (ELISAs) to measure immunoreactivity to ASPH, vimentin, and Large Acidic Ribonuclear Protein (RPLPO) as previously described [26,57]. (See Table 1 for antibody sources, characteristics, validations, and conditions of use. RPLPO served as a loading control due to its linear correlation with protein content [58].

### Western blot analysis

Western blot analysis was performed by fractionating protein (30μg samples) in 10% SDS-PAGE gels along with pre-stained molecular weight standards (Precision Plus Protein Dual Color Standards, Bio-Rad Laboratories, Hercules, CA, USA; Catalog #161-0374). The samples were electroblot transferred to polyvinylidene difluoride (PVDF) membranes (Bio-Rad Laboratories, Hercules, CA, USA) and incubated with SuperBlock-TBS (Thermo Fisher, Bedford, MA USA) to mask non-specific binding sites. The membranes were then incubated with primary antibodies overnight at 4°C with gentle platform agitation. Immunoreactivity was detected with horseradish peroxidase (HRP)-conjugated secondary antibody and Femto-chemiluminescence reagent (Thermo Fisher Scientific; Prod #34096). Immunoreactivity was imaged using the Intelligent Dark Box (FUJIFILM, Tokyo, Japan) and quantified with Fuji-Image-J software (U. S. National Institutes of Health, Bethesda, MD, USA). (The original, uncropped Western blot membrane can be found in a Supplementary File).

### Enzyme-linked immunosorbent assay (ELISA)

Triplicate 50ng aliquots of protein were adsorbed overnight to the bottom surfaces of Enzyme Immunoassay (EIA) Maxi sorp flat bottom 96-well plates (Thermo Fisher Scientific; Catalog #436110). Non-specific sites were masked with Superblock-TBS Thermo Fisher Scientific; Catalog #37535. Primary antibodies were incubated overnight at 4°C. After thorough rinsing of the wells, the samples were sequentially incubated with Horseradish Peroxidase (HRP) conjugated secondary antibody and Amplex Ultra Red fluorophore with TBS rinses between steps. Fluorescence intensity (Ex530nm/Em590 nm) was measured in a Spectra-Max M5 instrument. Negative controls included omitting the primary antibody, secondary antibody, or Amplex Ultra Red. The results normalized to RPLPO which served as a sample loading control [57].

### Immunocytochemistry

Cytospin preparations of cultured cells were generated using a Shandon Cytospin 3 Cytology Centrifuge with Rotor (Marshall Scientific, Hampton, NH, USA). Cell grown in 6-well plates were trypsinized and re-suspended to 0.5 × 10^6^ cells/mL in culture medium containing 0.5% FBS to prevent non-specific lysis. Approximately 0.5 × 10^5^ cells were centrifuged onto Plus-charged glass microscope slides at 600 rpm for 5 minutes at RT, and then immediately fixed in 10% neutral buffered formalin. Replicate slides were stained with Crystal violet (Thermo Scientific Chemicals, Haverhill, MA, USA) to examine cell morphology, or immunostained with the FB50 ASPH monoclonal antibody (Laboratory-made). Immunoreactivity was detected with the Impress peroxidase polymer detection reagents (Vector Laboratories, Newark, CA) and Diaminobenzidine (DAB) as the chromogen according to the manufacturer’s protocol. The immunostained cells were lightly counterstained with Gill’s Hematoxylin 2, dehydrated through graded ethanol solutions, and preserved under cover glass with Cytoseal 60 mounting media (Epredia; REF#8310-4).

### Motility assay

Directional motility was measured using the ATP Luminescence-Based Motility-Invasion (ALMI) assay [59]. These assays were constructed with blind well chambers (Neuro Probe, Gaithersburg, MD, USA) divided into upper and lower segments using 13mm diameter, 8-μm pore diameter polycarbonate membranes. Motility was measured using uncoated membranes. Motility and invasion were measured using membranes that were coated with a thin layer of 1% Geltrex (A1413201, Invitrogen, Carlsbad, CA USA). Just prior to use, Geltrex coating was achieved by incubating the polycarbonate membranes in solution at 37°C for 30 minutes, and then air-drying them at room temperature for 15 minutes under a cell culture hood. The motility/invasion assay was assembled by placing culture medium (200μL) containing 1%FBS as the trophic factor in the blind well (lower part of the chamber below the membrane), and 1.0 × 10^5^ viable cells in 100μl of serum-free medium in the upper chamber. The assays were incubated for 1h at 37°C in a standard CO_2_ cell culture incubator. Results were analyzed by measuring ATP luminescence in the cells remaining in the upper chamber (non-migrated), distributed on the undersurface of the membrane (migrated adherent), and distributed in the lower chamber (migrated non-adherent). ATP content with measured with ATP-Lite reagents (Perkin-Elmer, Waltham, MA, USA) as previously described [59]. Luminescence was quantified in a Top count NXT Microplate Scintillation and Luminescence Counter (Packard). The percentages of non-motile, motile adherent, motile non-adherent cells in replicate assays were calculated and used for statistical analysis.

### Invasion assays

The effects of DOX, SMI-1182 and the dual treatments with DOX+SMI-1182 on cell invasion were evaluated using the 96-well 3D Spheroid Basement Membrane Extract (BME) Cell Invasion BME assay kit (R&D Systems, Minneapolis, MN, USA, 3500-096-K). The assays were constructed by first allowing spheroids to form by seeding each well with 3000 viable cells in 50μL of medium containing 5% FBS and 1x Spheroid formation Extracellular Matrix (ECM). After 3 minutes of low-speed swinging bucket centrifugation (200 × g), the cultures were maintained for 72h in a standard 37°C, 5% CO_2_ incubator. To assess invasive growth, the spheroid cultures were overlaid with 50μL commercially supplied (kit) invasive matrix supplemented with DOX, SMI-1182, DOX+SMI-1182, or vehicle and incubated for 7 days. At that experimental end-point, the cultures were imaged and spheroid dimensions and numbers were quantified using Image-J software (U. S. National Institutes of Health).

### Cell Transformation Assay

The CytoSelect^™^ 96-Well Cell Transformation Assay (Soft Agar Colony Formation) (Cell Biolabs, CBA-130) was used to assess the effects of the various treatments on CS cell transformation capacity. A 50μL base agar layer composed of 0.6% Agar in RPMI and 10% FBS was formed in each well of a 96-well flat-bottom plate. After 30 minutes incubation to enable solidification, a cell agar layer formed by first adding 75μL 0.6% agar/RPMI/10% FBS followed by 3000 CS cells in 25μL of medium. Negative controls excluded cells from the cell-agar layer. After 15 minutes to allow the cell-agar layer to solidify, the cultures were treated by the addition of 100μL of medium containing vehicle, DOX, SMI-1182, or DOX+SMI-1182 and incubated at 37°C for 7 days. After imaging and enumerating the colonies, the cells were lysed, labeled with CyQuant GR dye. Fluorescence was quantified in a Spectra-Max M5 (Ex485 nm/Em520 nm) as an index of anchorage-independent growth.

### Quantigene 2.0 RNA multiplex assay

Total RNA was extracted from 6-well CS cultures using QIAzol Lysis Reagent (Germantown, MD USA). Messenger RNA corresponding to ASPH, HIF-1α, Notch signaling molecules, and Insulin/Insulin-Like Growth Factor (IGF) networks were quantified with a custom Quantigene 2.0 Multiplex (QGP) Assay (Affymetrix Inc., Santa Clara, CA USA). HPRT1 served as the internal control gene. Cooperative hybridization and quantification were performed following the manufacturer’s protocol. Briefly, the assay allows for direct mRNA quantification using xMAP Luminex beads and reporter signal amplification. A working bead mix containing lysis mixture, blocking reagent, capture beads, and a 2.0 probe set was prepared and distributed in a 96-well format plate. Total RNA (1μg) was added to the sample wells and incubated overnight with the xMAP fluorescent beads. Sterile nuclease-free water was used as the background negative control. The samples were first incubated with a set of oligonucleotide probes (pre-amplifier, amplifier and bio-tinlabel), followed by streptavidin-conjugated R-Phycoerythrin (SAPE). The resulting fluorescent signals were detected with a Luminex MAGPIX instrument (Diasorin, Austin TX USA). MAGPIX calibration and verification standards were used throughout, ensuring the levels of SAPE fluorescence were proportional to RNA transcript abundance captured by the beads. After subtracting the probe-related background from the target Median Fluorescence Intensity (MFI), results were normalized to HPRT1.

### Data analysis

All assays were repeated at least 3 times with 3 or 4 technical replicates per sample. Results depict mean ± S.D. T-test, Analysis of Variance, and functional curve analyses were performed using GraphPad Prism 10.2 (GraphPad Software Inc., San Diego, CA, USA). CompuSyn software (Informer Technologies, Inc., USA) was used to calculate the Combination Index (60) for determining if dual treatment effects were additive or synergistic.

### Reagent sources

The ELISA MaxiSorp 96-well plates, Bicinchoninic Acid (BCA) reagents, Horseradish Peroxidase (HRP)-conjugated secondary antibodies, and Superblock (TBS) were purchased from Thermo-Fisher Scientific (Bedford, MA USA). The soluble fluorophores, Amplex UltraRed and 4-Methylumbelliferyl phosphate (4-MUP) were from Life Technologies (Carlsbad, CA, USA). All other fine reagents were purchased from CalBiochem/Millipore Sigma (Burlington, MA, USA), Pierce Chemical (Dallas, TX, USA), or Sigma-Aldrich Co. (St. Louis, MO, USA).

## Results

### Monotherapy effects of smi-1182 and dox on metabolic activity and cell viability

**Figure 1:**
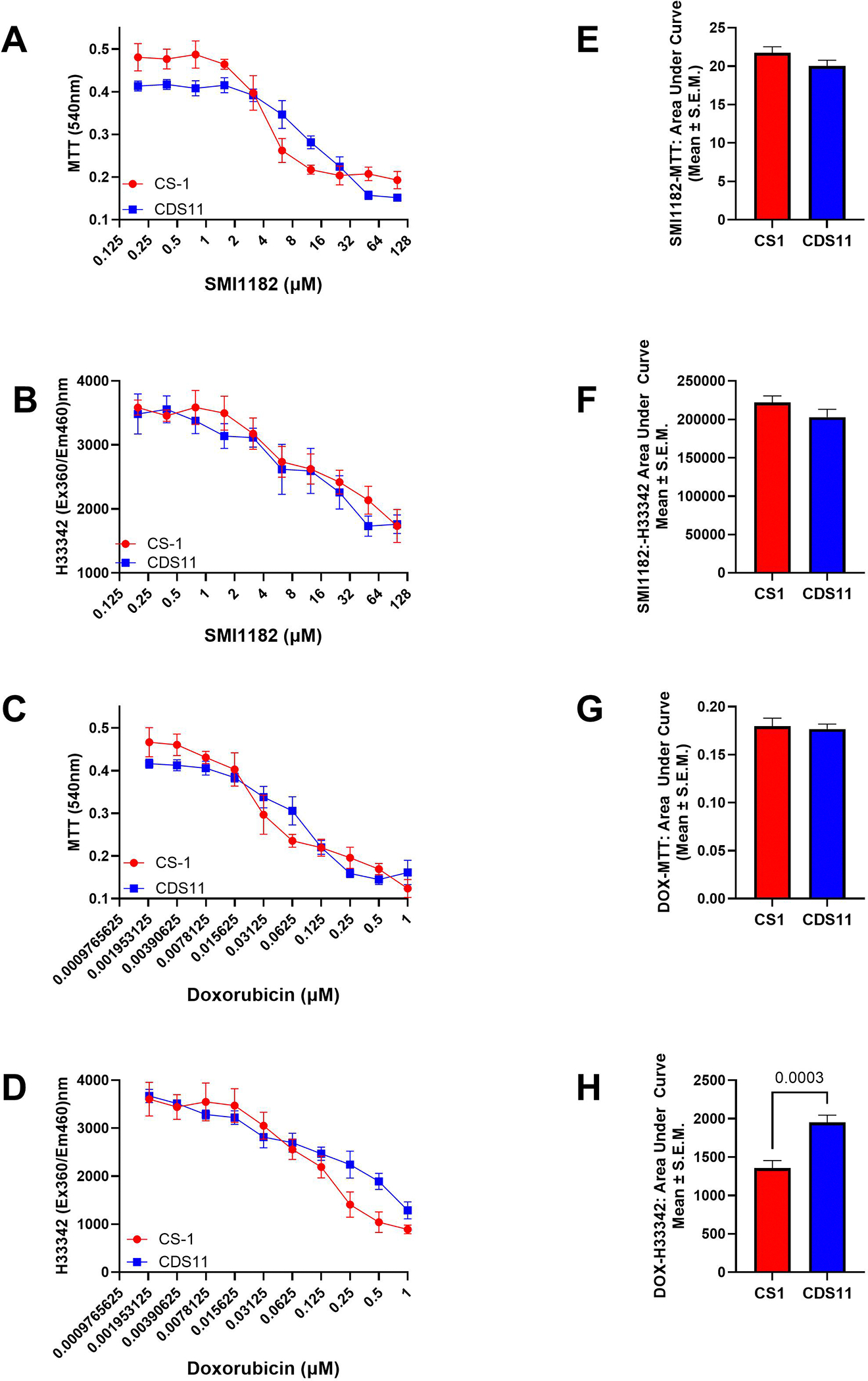
Dose effects of SMI-1182 and Doxorubicin (DOX) on Chondrosarcoma Cell MTT Activity and Viability. CS-1 and CDS11 human conventional chondrosarcoma cells were seeded into 96-well micro-cultures and treated with dose ranges of SMI-1182 or DOX for 48h. (A, C) MTT activity (absorbance) and (B, D) Hoechst H33342 fluorescence were measured in 4 replicate wells per plate. Panels E-H depict area-under-curve analyses. Data points and bar graphs show mean ± S.D. of results. Inter-group differences were compared by T-test. Significant p-values are shown.

CS-1 and CDS11 human conventional chondrosarcoma cells were evaluated for changes in MTT activity and H33342 fluorescence (index cell number) in 96-well cultures treated with dose ranges of SMI-1182 (0 to 100μM) or DOX (0, 0.05, 0.5, or 1.0 μM) for 48h (Figures 1A–1D). The MTT assay provides information about mitochondrial function, proliferation, and cell viability. However, under conditions of mild oxidative stress, MTT activity increases, potentially leading to erroneous interpretations about cellular responses, e.g, proliferation. To complement the MTT studies, the cells were also stained with H33342 dye which labels DNA and is linearly correlated with cell number. Dying cells detach from the plate and thus are not counted/measured in the assay. The cellular analyses were further enhanced by measuring G6PD release as a measure of cytotoxicity (see below). Together, these three assays enabled us to assess CS cellular responses to DOX and SMI-1182.

**Figure 2:**
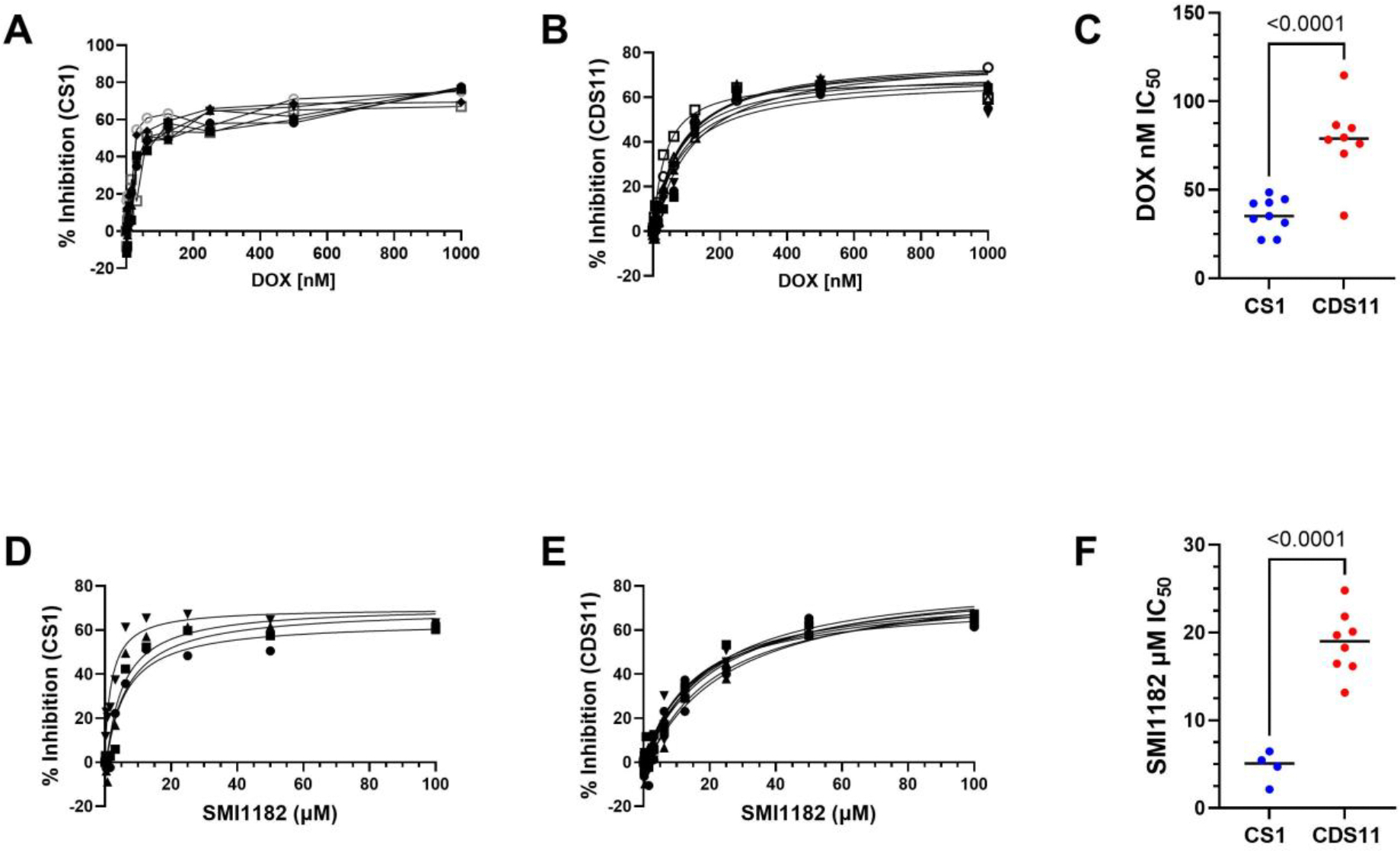
Comparative IC50 Analysis of SMI-1182 and DOX in CS-1 and CDS11 cells. (A,B,D,E) Curves show dose-dependent percent inhibition of viability and MTT activity relative to vehicle control (DMSO). (C, F) The graphs depict the aggregate results of IC50s calculated for each curve. Intergroup differences were compared by T-test (See Table below).

The dose-effect curves were compared by Area-Under-Curve (AUC) analysis (Figures 1E–1G). SMI-1182 and DOX treatments each caused dose-dependent reductions in MTT activity (Figures 1A and 1C) and H33342 fluorescence (Figures 1B and 1D) in CS-1 and CDS11 cells. The CS-1 and CDS11 dose-effect curves overlapped resulting in similar AUC calculation for SMI-1182 treatment (Figure 1E and 1F). However, the cellular responses differed at the higher doses of DOX such that H33342 fluorescence was more prominently reduced in CS-1 compared with CDS11 cells (Figure 1D). Correspondingly, the mean AUC for H33342 (viability) was significantly higher in CDS11 cells (p=0.0003) (Figure 1H). Further comparisons between the cell lines were made by calculating the 50% inhibitory concentrations (IC_50_) using GraphPad Prism, Version 10.4 (San Diego, CA, USA). The results demonstrated significantly higher IC_50_’s in CDS11 cells treated with either DOX (Figures 2A–2C) or SMI-1182 (Figures 2D–2F) (both p<0.0001). Together, these findings suggest that compared with CS-1, the CDS11 cells are more resistant to DOX- and SMI-1182-mediated metabolic dysfunction and cell loss.

### Impact of combination smi-1182 and dox therapy on metabolic activity, cell viability, and cytotoxicity

**Figure 3:**
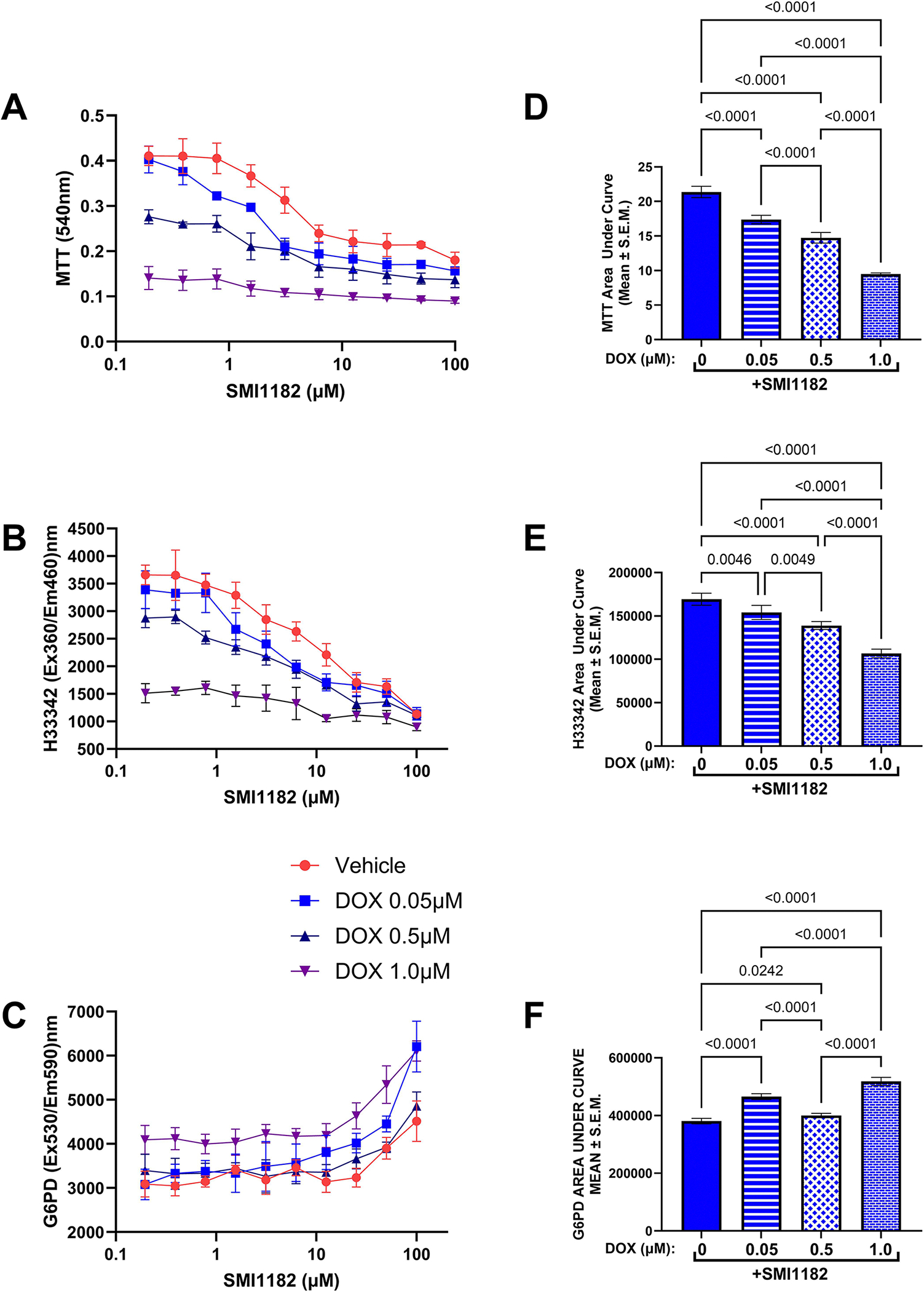
Combination therapy dose-effects (mean ± S.D.) on (A) MTT activity, (B) Hoechst H33342 fluorescence, and (C) cytotoxicity (G6PD release assay) in CS-1 cells. (E-F) Bar graphs show area-under-curve analysis results after 48h treatment with a dose range of SMI-1182 and 4 distinct levels of DOX (0, 0.05μM, 0.5μM and 1μM). Treatment effects were analyzed by one-way ANOVA with post hoc Tukey tests. P-values are shown within the graphs.

**Figure 4:**
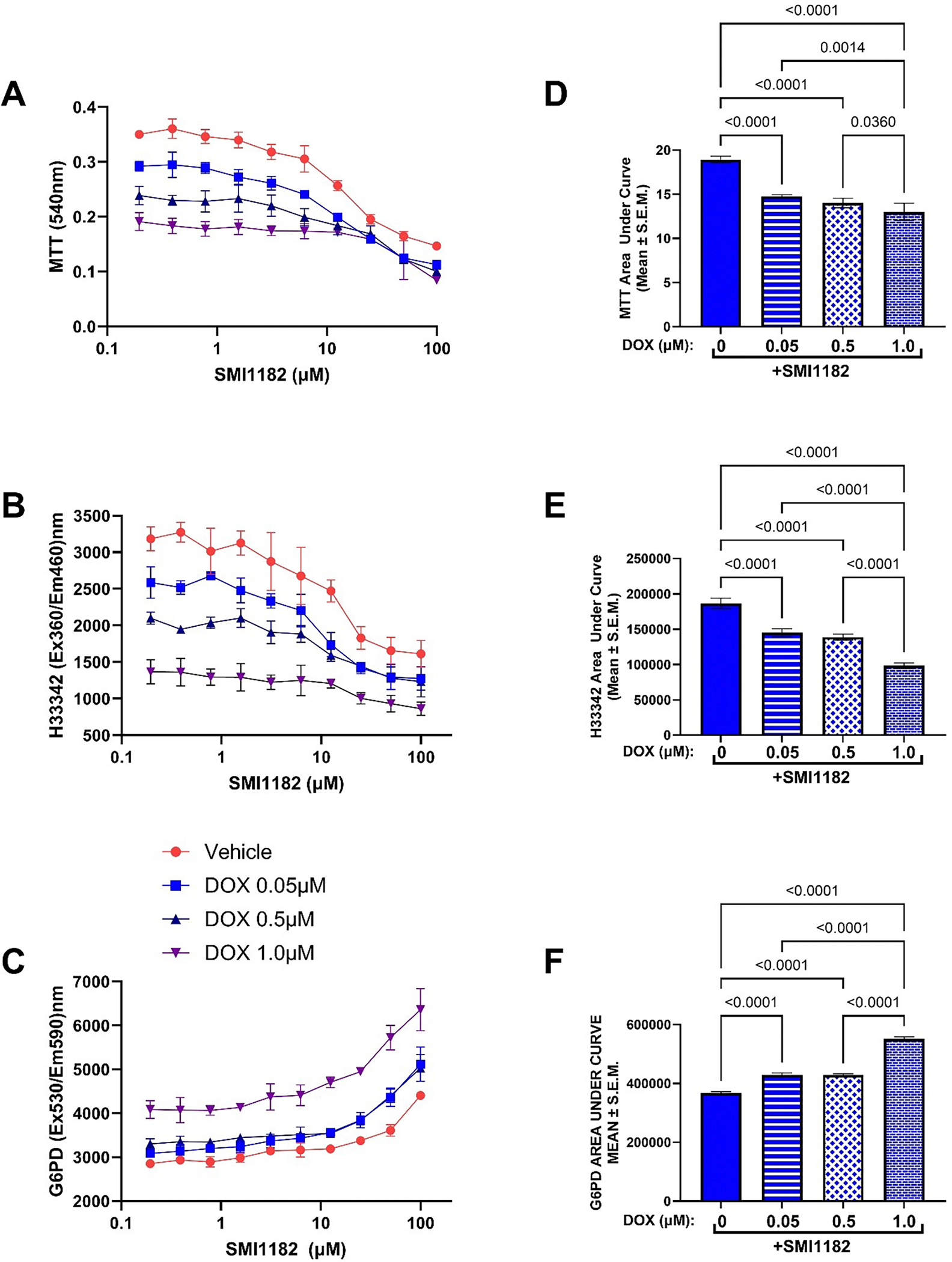
Combination therapy dose-effects (mean ± S.D.) on (A) MTT activity, (B) Hoechst H33342 fluorescence, and (C) cytotoxicity (G6PD release assay) in CDS11 cells. (D-F) Bar graphs show area-under-curve analysis results after 48h treatment with a dose range of SMI-1182 and 4 distinct levels of DOX (0, 0.05μM, 0.5μM and 1μM). Treatment effects were analyzed by one-way ANOVA with post hoc Tukey tests. P-values are shown within the graphs.

CS-1 and CDS11 cells were evaluated for changes in MTT activity (Figure 3A and 4A), H33342 fluorescence (Figure 3B and 4B), and G6PD release (measure of cytotoxicity) (Figure 3C and 4C) in 96-well cultures treated with SMI-1182 (0 to 100μM) combined with DOX (0, 0.05, 0.5, or 1.0 μM) for 48h. The dose-effect curves were compared by area-under-curve analysis (Figures 3D–3F and 4D–4F). For CS-1 cells, MTT activity and H33342 fluorescence declined and G6PD release increased with increasing dose of SMI-1182, with the largest responses for MTT and H33342 occurring above 1.0μM SMI-1182, and for G6PD, the sharpest increased occurred with 10μM or higher SMI-1182. DOX additions caused significantly greater declines in MTT and H33342 and increases in G6PD release relative to SMI-1182 treatment alone, and the effect sizes were generally larger at the higher doses of DOX (Figure 3). SMI-1182 doses above 10μM rendered low-dose DOX as cytotoxic as high-dose DOX. Also, at the highest DOX dose, MTT and viability (H33342) were uniformly low, and cytotoxicity was elevated across the full dose range of SMI-1182.

CDS11 cells exhibited the most striking declines in MTT (Figure 4A) and H33342 (Figure 4B) at or near 10μM SMI-1182 versus 1.0μM for CS-1 cells. In contrast, the curves corresponding to G6PD release were similar for CDS11 (Figure 4C) and CS-1 (Figure 3C) cells. AUC analysis of the aggregate SMI-1182+DOX dose effects revealed progressive and significant declines in MTT (Figure 4D) and H33342 (Figure 4E), and increases in G6PD release, i.e, cytotoxicity (Figure 4F) from 0μM to 0.05μM, and then 1.0μM DOX. However, there were no significant differences in the effects of 0.05μM versus 0.5μM DOX when combined with SMI-1182.

To better understand the differences between CS-1 and CDS11 cells regarding the dual treatment responses, the AUC analyses for MTT (Supplementary Figure S1A), H33342 (Figure S1B), and G6PD release (Figure S1C) were compared by Two-way ANOVA (Table 2). Significant effects of CS cell type, i.e, tumor grade, were observed for MTT, and significant effects of DOX dose and CS Grade × DOX dose interactions were observed for MTT, H33342, and G6PD. The corresponding graphs shown in Figure S1 depict the results of post hoc multiple comparisons tests. The findings were that CDS11 cells had significantly lower mean MTT AUC calculations relative to CS-1 at the 0 and 0.05μM DOX doses, but higher levels at the highest DOX dose (1.0μM) (all p<0.0001). The H33342 comparisons revealed significantly higher overall mean viability in the absence of DOX, but reduced viability only at the 0.05μM DOX level in CDS11 cells Figure S1B). G6PD release following treatment with SMI-1182+ 0μM or 0.05μM DOX was significantly lower in CDS11 versus CS-1 cells, but relatively at higher co-treatment doses of DOX (Figure 1C).

**Figure 5:**
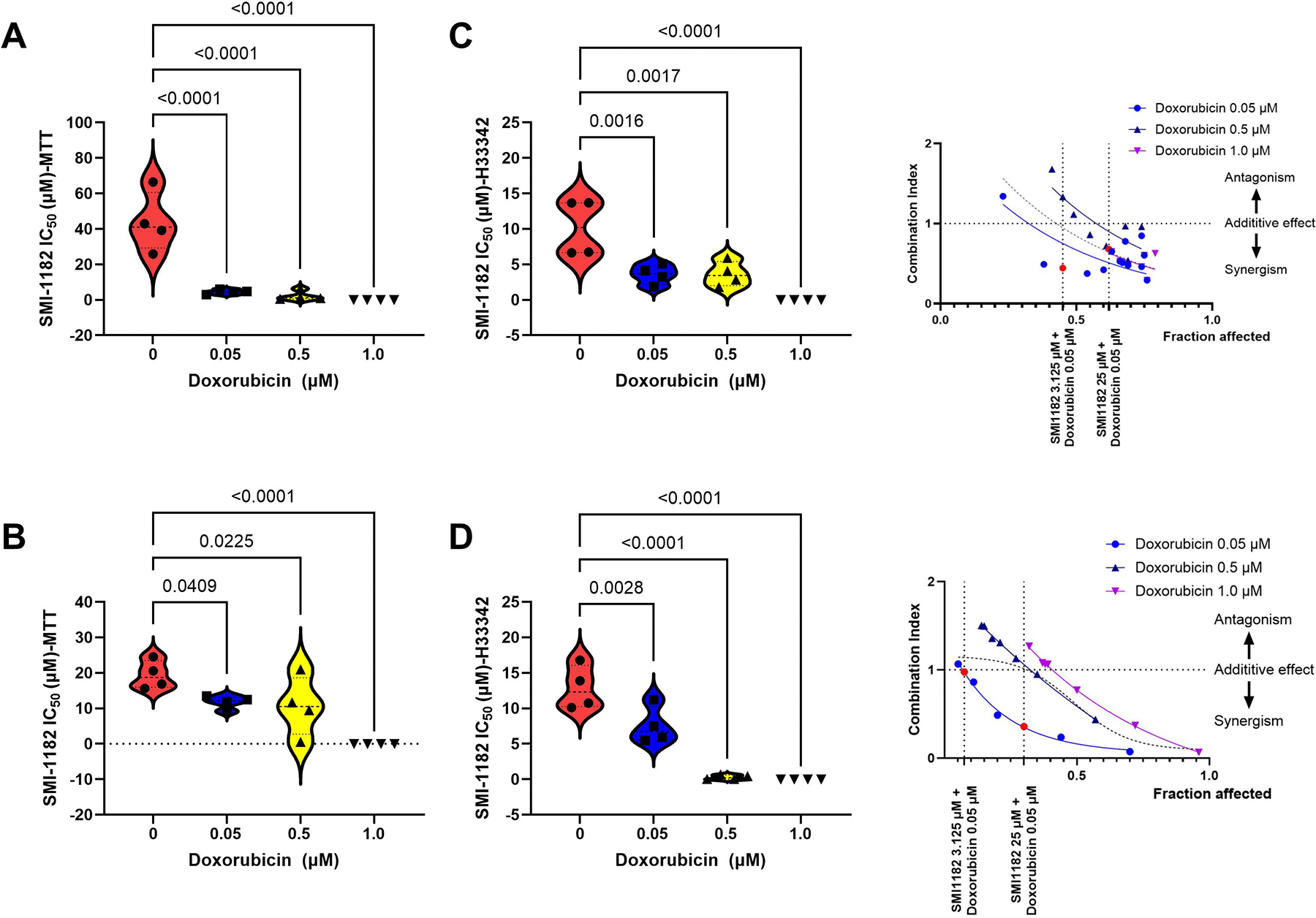
Combination therapy with SMI-1182 and DOX Adversely Impacts CS Cell Metabolic and Viability Functions and Increase Cytotoxicity. The cells were treated with a dose range of SMI-1182 (0–25μM), and 0μM, 0.05μM, 0.5μM, or 1.0μM DOX for 48h. Violin plots depict the distribution of results with the median (horizontal bars) and 95% confidence interval limits for 4 separate IC50 calculations corresponding to MTT activity (mitochondrial function) in (A) CS-1 and (B) CDS11 cells, H33342 fluorescence (viability) in (C) CS-1 and (D) CDS11 cells. The graphs were generated with Graphpad Prism 10.4 and analyzed by one-way ANOVA (See Table 2). Post hoc test results are shown in the graph panels. (E, F) CompuSyn software analysis of combination SMI-1182 and DOX dose-effects on cytotoxicity (G6PD release) in (E) CS-1 and (F) CDS11 cells. The horizonal bars correspond to the IC50s. Values above the bar reflect antagonistic effects. Values at the bar reflect additive effects. Values below the bar correspond to synergistic effects.

Further comparisons between CS-1 and CDS11 responses to combination SMI-1182 + DOX were made by calculating the mean IC50s corresponding to MTT activity (Figures 5A and 5B) and cell viability (H33342 fluorescence) (Figures 5C–5D). One-way ANOVA tests demonstrated significant inter-group differences in the mean IC50 associated with MTT in CS-1 (F=23.4; p<0.0001) and CDS11 (F=11.29, p=0.0008), and H33342 in CS-1 (F=13.54, p=0.0004) and CDS11 (F=37.55, p<0.0001). The post hoc multiple comparisons tests (See Figures 5A–5D) demonstrated significantly lower mean IC50s for both MTT and H33342 in cultures treated with dual therapy, i.e, SMI-1182+DOX (all levels) compared with SMI-1182 monotherapy, i.e, without DOX. In CS-1 cultures, the dual treatment IC50 was sharply and similarly reduced by co-treatment with varying doses of DOX. In CDS11 cultures, the declines in IC50 were more gradual and DOX-dose dependent.

To determine if the potentiating effect of SMI-1182 on DOX was additive or synergistic, we used CompuSyn software (Informer Technologies, Inc., USA) for cytotoxicity (G6PD release) to compute the Combination Index (CI) based on the Chou-Talalay method for drug combination [60]. Graphs corresponding to the results of the CompuSyn analysis of G6PD release (cytotoxicity) in CS-1 and CDS11 cells are shown in Figure 5E and 5F. In CS-1 cells, combination therapy at the lowest (0.05μM) and highest (1.0μM) DOX doses had predominantly synergistic effects on cytotoxicity, whereas the intermediate DOX dose (0.5μM) produced mixtures of synergistic and antagonistic effects on cytotoxicity (Figure 5E). In CDS11 cells, the CI curves also showed largely synergistic effects of combination therapy at the lowest and highest DOX concentrations but mixed antagonistic and synergistic responses to SMI-1182+ 0.5μM DOX (Figure 5F). Quantitative analysis of the results revealed CIs of less than 1.0, i.e, synergistic responses in CS-1 and CDS11 cells exposed to 3.125μM SMI-1182 up to 25μM SMI-1182 and DOX= 0.05μM or 1.0μM, indicating synergistic effects (Figure 5E).

### DOX and SMI-1182 Effects on CS Cell Morphology and ASPH Expression

**Figure 6:**
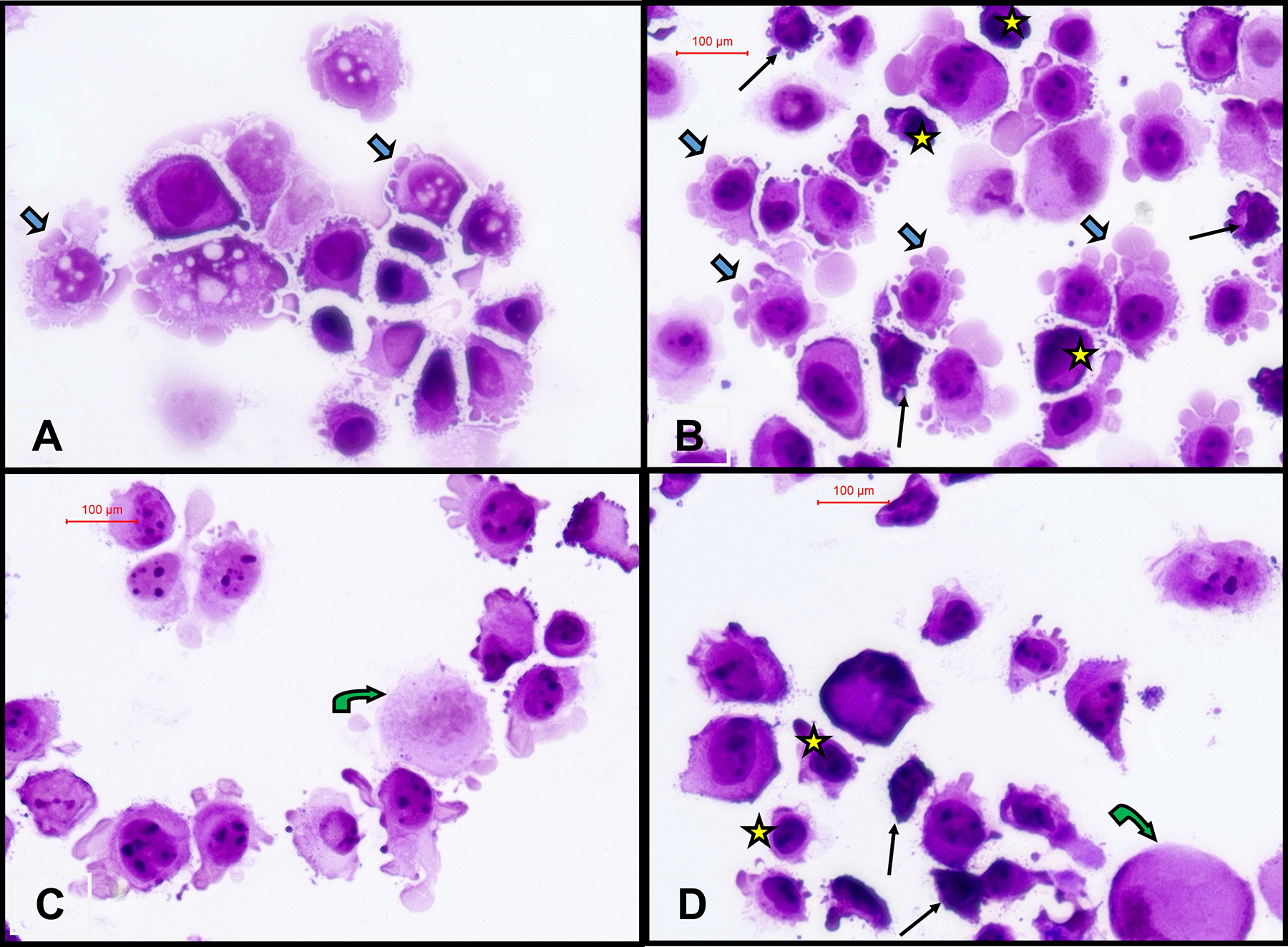
Cytomorphological effects of (A) Vehicle, (B) SMI-1182, (C) DOX, and (D) SMI-1182+DOX. Cytospin preparation after 48h treatment were fixed in formalin, stained with Crystal violet, and examined by light microscopy. (A) Control cells exhibit conspicuous variation in cell size, nuclear vacuolation or condensation and moderate cell surface blebbing (arrowheads). (B) SMI-1182 treatment prominent cytoplasmic blebbing (arrowhead), nuclear condensation (asterisks) or enlargement, and cellular necrosis (arrows). (C) DOX treatment increased nucleolar prominence, blebbing, and apoptosis which was evidenced by nuclear fragmentation and loss (D) SMI-1182+DOX treatment yielded combined effects of the separate treatments, but notably increased nuclear condensation and necrosis.

Cytospin preparations of vehicle-treated CS cells stained with Crystal Violet exhibited pleomorphic polygonal morphology with high nuclear: cytoplasmic ratios, but variability in cell size, nuclear condensation, and cytoplasmic vacuolation (Figure 6A). DOX (0.05μM) increased cell size, shape variability, nuclear condensation, apoptosis, and cellular fragmentation (Figure 6B). SMI-1182 altered nuclear morphology, enhanced nucleoli, and caused prominent surface blebbing and cellular enlargement (Figure 6C). Combined SMI-1182 and DOX treatments produced overlapping cytomorphological changes corresponding to the effects of DOX and SMI-1182, in addition to increased nuclear fragmentation and condensation and cellular enlargement (ballooning) (Figure 6D).

**Figure 7:**
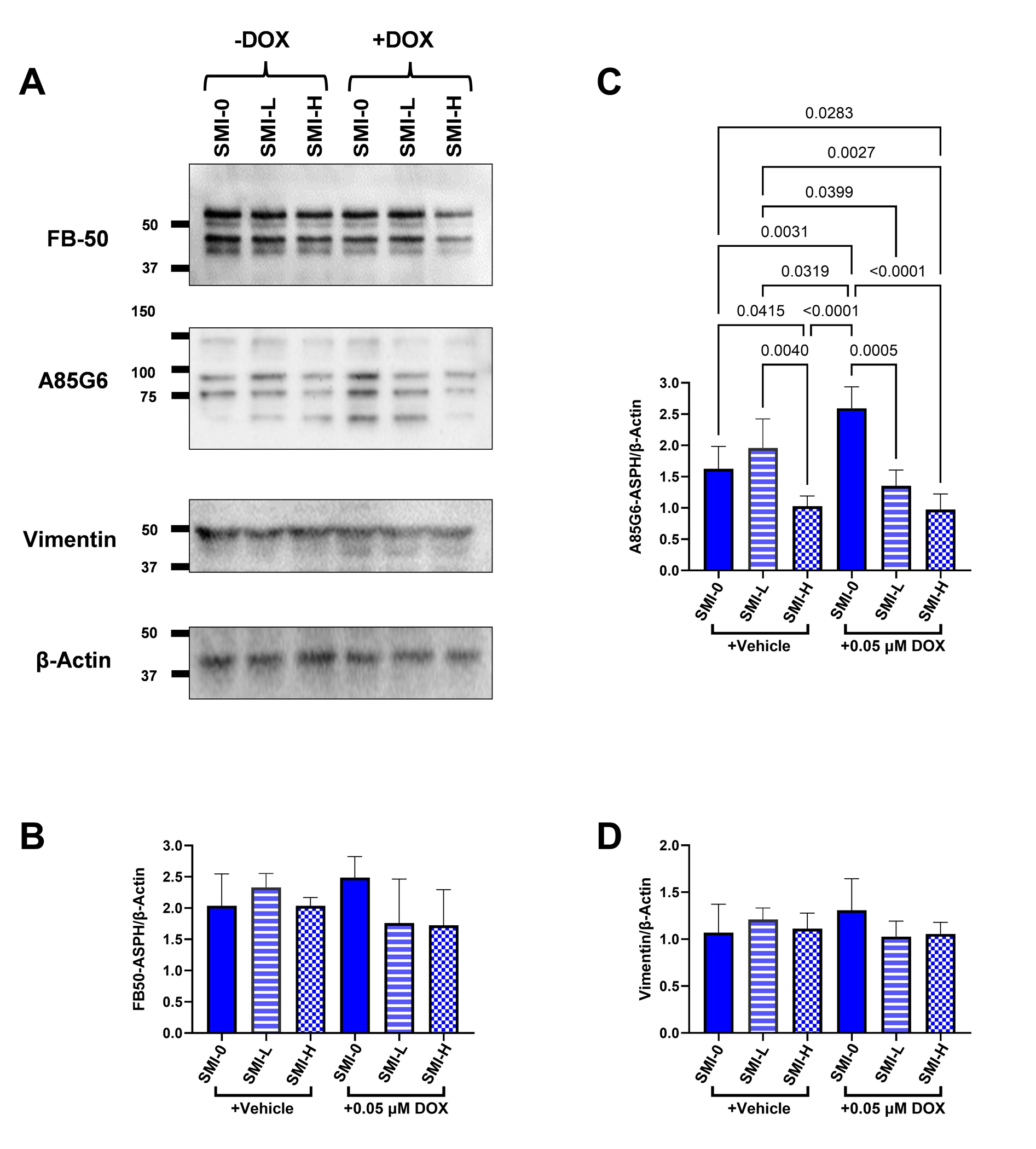
Western blot analysis of ASPH after 48h treatment with SMI-1182 (SMI), DOX, or both. CS-1 cells were treated with vehicle (SMI-0μM), 3.125μM (SMI-L) or 25μM (SMI-H), DOX (0.05μM) or SMI-(0, L, H)+DOX. (A) Western blots were probed with FB50 or A85G6 monoclonal antibodies to the N-terminal or C-terminal region of ASPH, Vimentin, or β-Actin (loading control). FB50 also detects Humbug, which is abundantly expressed in malignant neoplastic cells. (B-D) Graphs depict the Mean ± S.D. of Image-J quantified results from 3 Western blot studies with the levels of immunoreactivity normalized to β-actin. One-way ANOVA tests were only significant for A85G6-ASPH. The significant (p<0.05) post hoc multiple comparisons results are shown in Panel C.

Western blot analysis and ELISA were performed with two ASPH monoclonal antibodies, FB50 and A85G6. FB50 binds to the N-terminal region of ASPH which overlaps with the sequence for Humbug, a truncated form of ASPH lacking the C-terminal catalytic domain [61]. A85G6 binds to the C-terminus of ASPH which contains a unique catalytic domain for Notch and Jagged hydroxylation [61]. Besides ASPH, the samples were probed for Vimentin as a positive control and β-actin or RPLPO as a loading control for Western blotting or ELISA, respectively. Western blot signals were quantified with Image-J software (U. S. National Institutes of Health) and ELISA results were measured in a Spectra-Max M5 microplate reader (Molecular Devices, Sunnyvale, CA, USA). Comparisons were made among CS cells treated with Vehicle (control; SMI-0), low-dose SMI-1182 (3.125 μM; SMI-L), high-dose SMI-1182 (25 μM; SMI-H), DOX (0.05 μM), SMI-L+DOX, and SMI-H+DOX. Example Western blots are shown in Figure 7A, and the image analysis results from three studies are shown in Figures 7B–7D.

Irrespective of treatment, CS-1 cells exhibited similarly high levels of FB50-ASPH immunoreactivity by Western blot analysis, corresponding with the negative ANOVA test result obtained with the image analysis data (F=1.309; N.S.) (Figure 7B). In contrast, the Western blot signals detected with A85G6-ASPH varied with treatment such that the highest levels were observed in DOX-treated cultures, and lowest in SMI-H, with or without DOX (Figure 7A). ANOVA test of the image analysis results demonstrated significantly higher levels of A85G6-ASPH in DOX compared with all other treatments, and significantly lower levels of A85G6-ASPH in SMI-H ± DOX and SMI-L+DOX relative to all other treatments (F=11.05; p=0.0004) (Figure 7C). Note that the FB50-ASPH and A85G6-ASPH Western blot signals depict characteristically fragmented/cleaved protein products. Vimentin immunoreactivity was similarly abundant across the different treatments (F=0.73; N.S.) (Figures 7A–7D).

**Figure 8:**
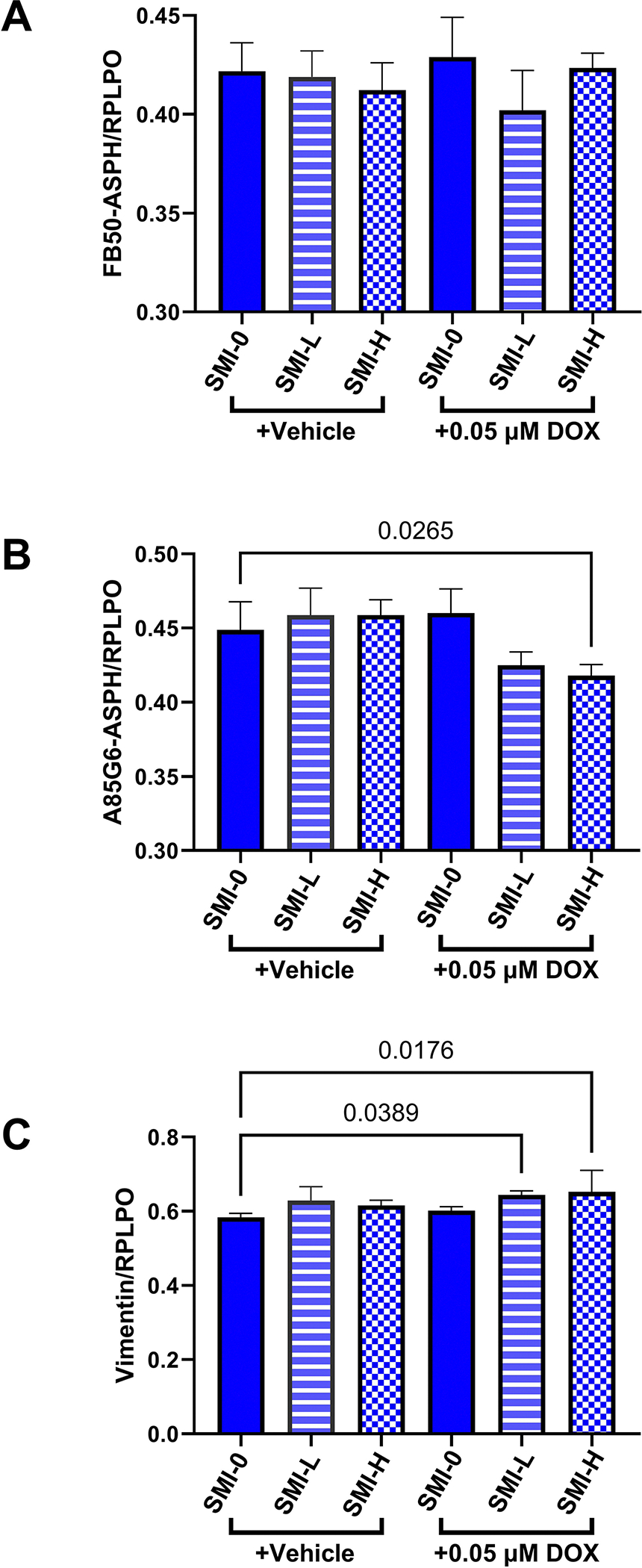
ELISA results demonstrating the effects of 48h treatment with SMI-1182, DOX, or SMI-1182+DOX on (A) FB50-ASPH, (B) A85G6-ASPH, and (C) vimentin. CS-1 cells were treated with vehicle, 3.125 or 25 μM SMI-1182, DOX (0.05 μM) or SMI-1182 + DOX. ELISA results were normalized to RPLPO. Graphs depict the mean ± S.D. of ELISA results obtained from 4 replicate cultures. Inter-group statistical comparisons were made one-way ANOVA with post hoc Tukey tests of significance.

The results obtained by ELISA largely confirmed the findings by Western blot analysis. The data were analyzed by one-way ANOVA and the post hoc tests were focused on treatment effects relative to control (Vehicle; SMI-0). Corresponding with the Western blot results, no significant treatment effects were observed for FB50-ASPH expression (F=1.522; N.S.) (Figure 8A). In contrast, significant inhibitory effects of SMI-L+DOX and SMI-H+DOX were detected for A85G6-ASPH (F=6.95; p=0.0009) (Figure 8B). In contrast to the Western blot results, ELISA studies detected increased Vimentin immunoreactivity in both SMI-L+DOX and SMI-H+DOX treated cells relative to control (F=3.075; p=0.0353).

**Figure 9:**
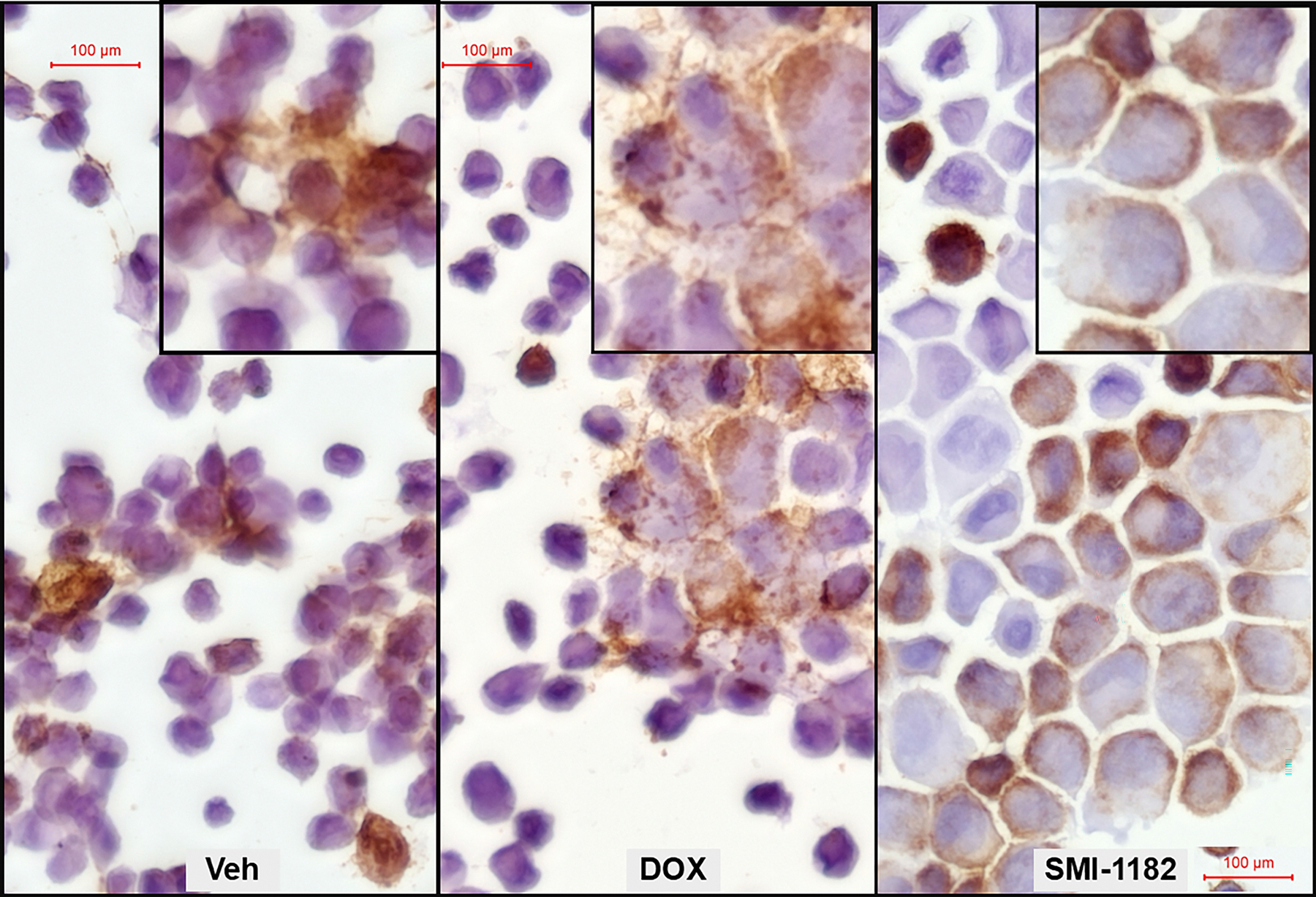
DOX and SMI-1182 alter CS cellular distributions of ASPH immunoreactivity. CS-1 cells treated with vehicle (Veh), 0.05 μM DOX (DOX), or 3.125 μM SMI-1182 (SMI-1182) for 48h, were harvested to generate cytospin preparations, fixed in 10% buffered formalin, and immunostained with the FB50-ASPH antibody. Immunnoreactivity was detected with the ImPRess kit with DAB as the chromogen (brown), followed by light Hematoxylin counterstaining (blue). Vehicle treated cells show surface, cytoplasmic and perinuclear FB50-ASPH immunoreactivity. DOX treatment resulted in conspicuous bead-like fibrillar labeling of cell surface processes, particularly in larger tumor cells (see inset). Pyknotic/shrunken cells with condensed nuclei (pre-necrotic or apoptotic) exhibit nuclear staining no FB50-ASPH immunoreactivity. SMI-1182 treatment resulted in FB50-ASPH immunoreactivity localized inside the plasma membrane or perinuclear rather than the cell surface. All images were obtained at 400x magnification. Scale bars are displayed. Insets show higher magnification images of FB50-ASPH-positive labeling.

Immunocytochemical staining of CS-1 cells with the FB50-ASPH antibody revealed surface, cytoplasmic, and perinuclear immunoreactivity in vehicle-treated cultures, and prominently increased fibrillar and beaded cell surface and pericellular labeling after DOX treatment (Figure 9). SMI-1182 treatment resulted in ASPH immunoreactivity tightly localized along the inner subjacent region of the plasma membrane rather than on the outer membrane surfaces (Figure 9).

### Cell Migration, Invasion and Colony Formation Studies

**Figure 10:**
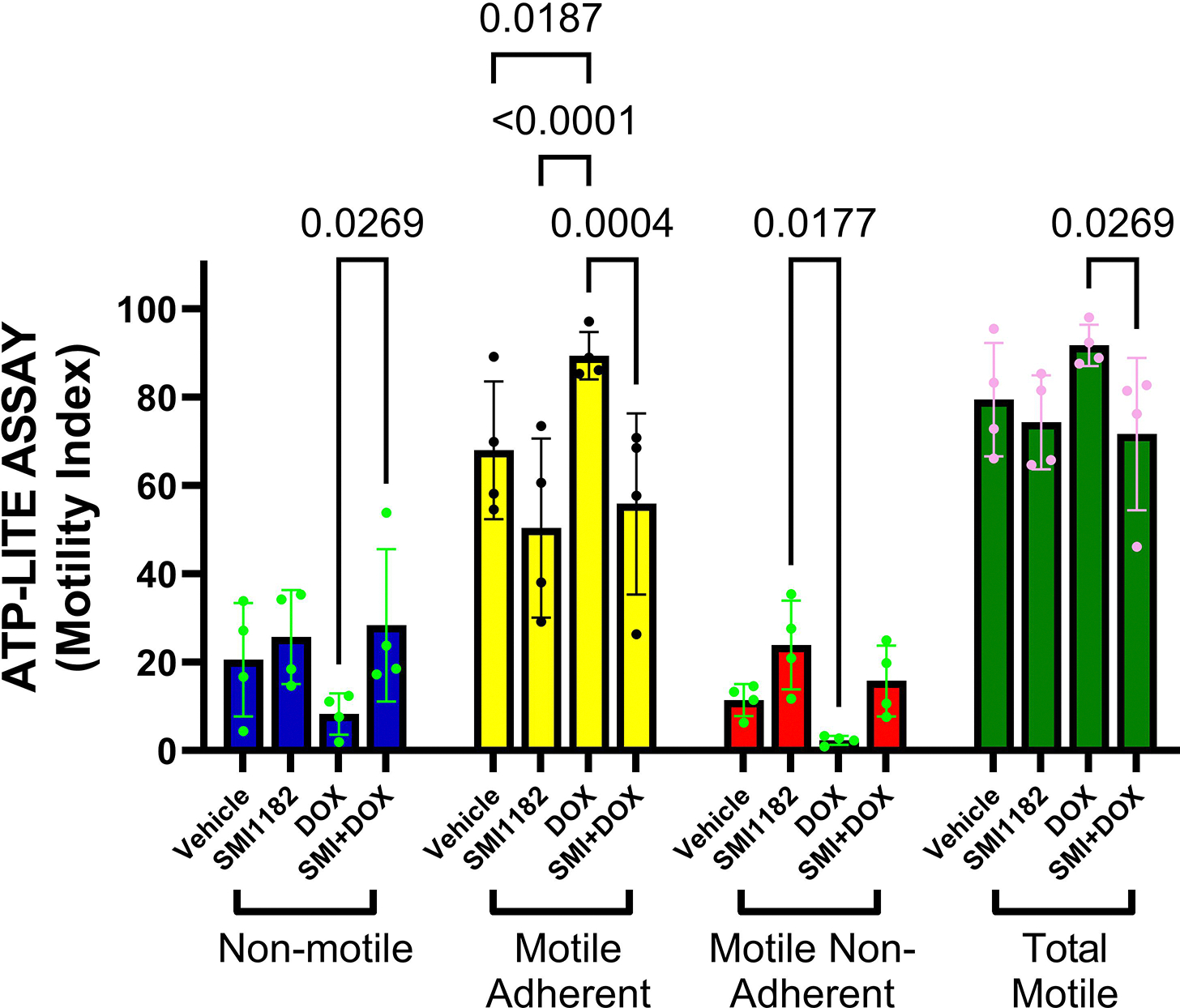
Effects of SMI-1182 and DOX on Directional Cell Motility and Adhesion. CS-1 cells were treated for 48h, then transferred to a Boyden chamber-type apparatus containing uncoated 8 μM pore diameter polycarbonate filters. Cell migration was allowed to proceed for 2h in a standard cell culture incubator. The ATP Lite assay was used to measure the motility index, i.e, the percentages of non-motile (remaining on the upper membrane surface), motile adherent (migrated through the pores but still adherent to the undersurface of the membrane), motile non-adherent (migrated to the bottom well of the chamber), and total motile (motile adherent+motile non-adherent). Results were analyzed by ANOVA with post-hoc multiple comparisons. Significant p-values are displayed.

The effects of 3.125μM SMI-1182, 0.05μM DOX and SMI-1182+DOX on directional motility were evaluated in CS-1 cells using the ATP-Lyte Motility assay with uncoated polycarbonate membranes. Two-way ANOVA revealed significant interactive effects between treatment and cell migration (F=4.474; p=0.0003) (Table 3). The post hoc multiple comparisons tests demonstrated that DOX significantly reduced the mean percentages of non-motile and motile non-adherent cells but increased the percentages of motile adherent and total motile populations (Figure 10).

**Figure 11:**
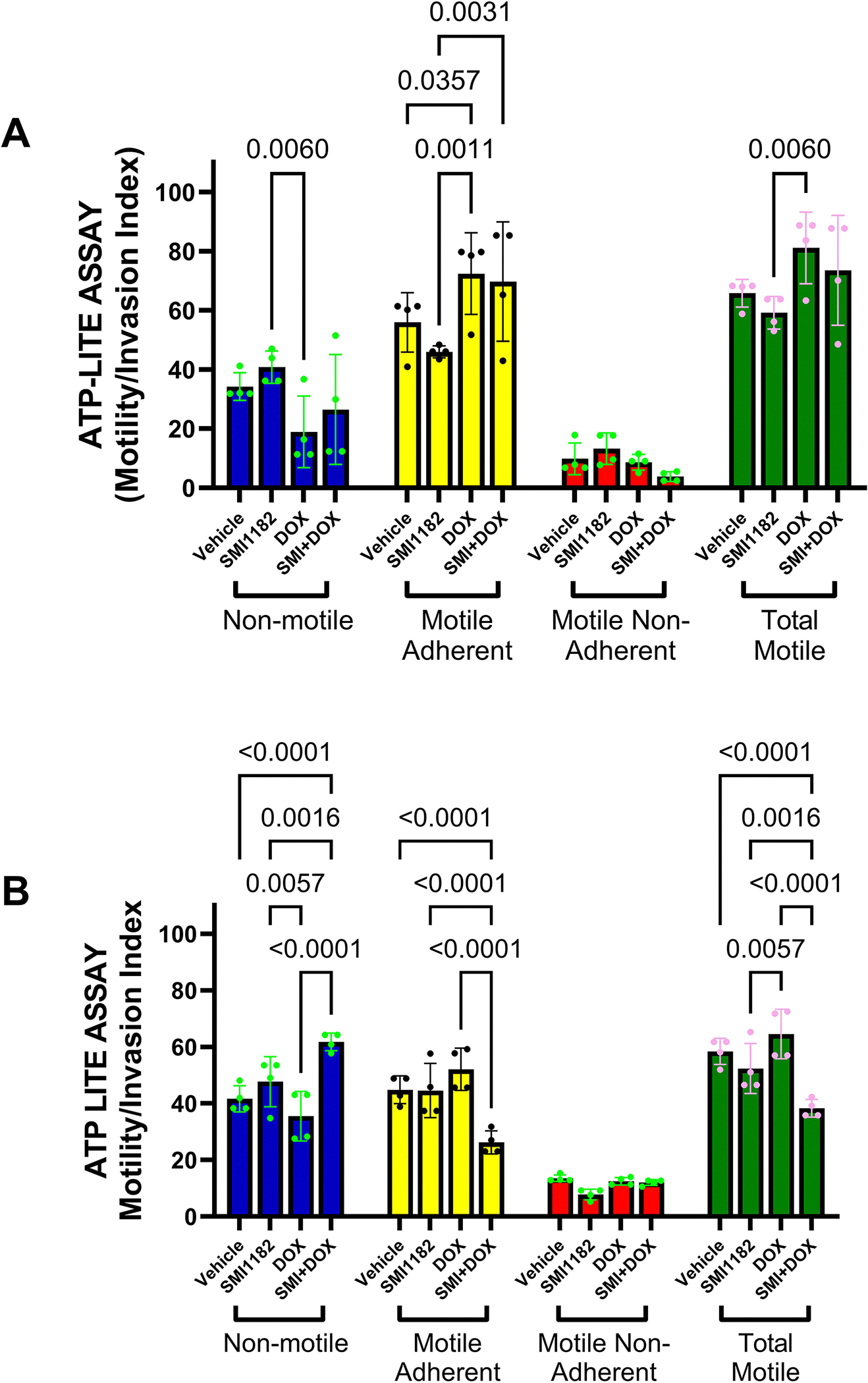
Effects of SMI-1182 and DOX on Directional Cell Motility and Adhesion. (A) CS-1 and (B) CDS11 cells were treated for 48h with vehicle, SMI-1182, DOX, or SMI-1182+DOX, then transferred to a Boyden chamber-type apparatus containing Geltrex-coated 8 μM pore diameter polycarbonate filters. Cell migration/invasion was allowed to proceed for 2h in a standard cell culture incubator. The ATP luminescence measured the motility/invasion index, i.e. the percentages of non-motile (remaining on the upper membrane surface), motile adherent (migrated through the pores but still adherent to the undersurface of the membrane), motile non-adherent (migrated to the bottom well of the chamber), and total motile (motile adherent+ motile non-adherent). Results were analyzed by ANOVA with post-hoc multiple comparisons. Significant p-values are displayed.

Further studies of CS-1 and CDS11 cells included GelTrex-coated membranes to simultaneously assess invasion of extracellular matrix (Figure 11). Two-way ANOVA detected significant treatment × motility interactive effects in CS-1 cultures (F=3.732; p=0.0013) and significant treatment (F=3.355; p=0.021) and treatment × motility interactions in CDS11 cells (F=13.08; p<0.0001) (See Table 3). Post hoc tests showed that the mean percentage of non-motile cells was increased, and the percentages of motile-adherent and total migrated cells were decreased in SMI-1182 compared to DOX treatment in CS-1 cultures (Figure 11A). In addition, the mean percentage of migrated-adherent cells was significantly reduced in SMI-1182 compared with SMI-1182+DOX treatment (Figure 11A). DOX also increased the percentage of motile-adherent cells relative to vehicle.

In CDS11 cultures, the dominant effects on motility and invasion were associated with dual SMI-1182+DOX treatment (Figure 11B). SMI-1182+DOX treatment resulted in significantly increase percentages of non-motile cells and reduced percentages of motile-adherent and total motile populations relative to the other three groups. In addition, DOX treatment reduced the mean percentage of non-motile cells and increased the percentage of total motile cells relative to SMI-1182. In essence, for CS-1 cells, SMI-1182 reduced directional cell motility and invasion relative to DOX monotherapy or when combined with low-dose SMI-1182. The responses in CDS11 cells differed from CS-1 in that the main effects were centered around significantly higher percentage of non-motile and lower percentages of motile adherent and total motile cells in cultures associated with dual SMI-1182+DOX treatments, suggesting that SMI-1182 may counteract the pro-invasive effects of DOX in this cell line.

**Figure 12:**
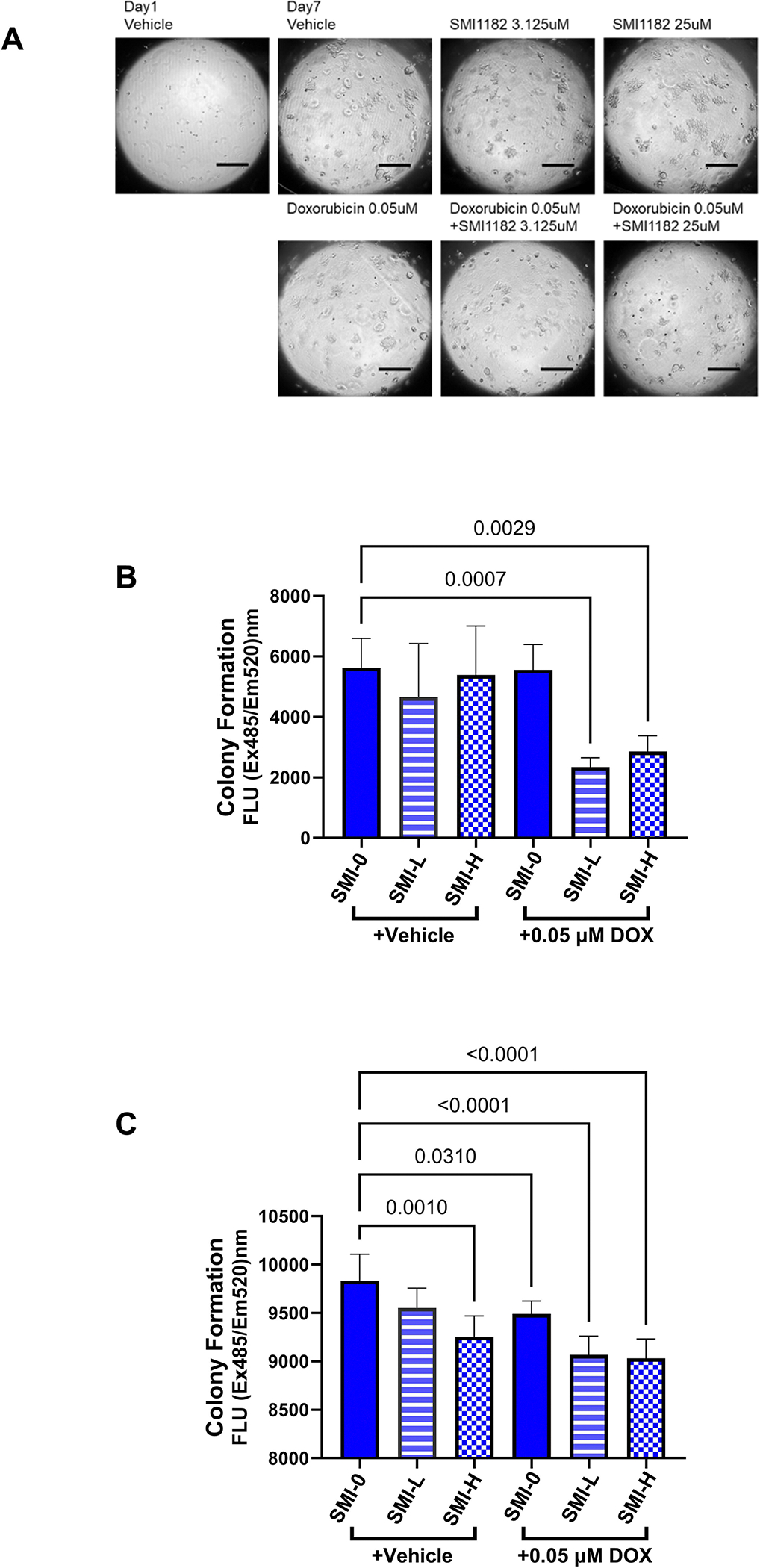
Soft Agar Colony Formation Assay. Anchorage-independent growth was assessed using the colony forming assay. (A) Representative images of the initial (Day 1) and final (Day 7) appearances of colony formation in cultures treated with Vehicle (DMSO), low (3.125 μM) or high (25 μM) SMI-1182, DOX (0.05 μM), or DOX+low- or high-dose SMI1182. Scale magnification bars (200 μm) are displayed. Colony formation was quantified in (B) CS-1 and (C) CDS11 cultures using the CyQuant assay. Inter-group comparisons were made relative to control (Vehicle) by one-way ANOVA with the Tukey post hoc multiple comparisons test. Significant differences from control are displayed.

Colony formation assays assessed the effects of SMI-L, SMI-H, DOX, SMI-L+DOX, and SM-H+DOX after 7 days treatment. Colony formation was visualized by light microscopy and quantified using CyQuant assay kit reagents. Example images are shown in Figure 12A. After 1 day in culture, colony formation was scant. After 7 days, Vehicle-, SMI-L, SMI-H, and DOX treatments yielded similarly abundant colony formation, whereas dual treatment with SMI-L+DOX or SMI-H+DOX substantially reduced colony formation relative to vehicle (Figure 12A). For both CS-1 (Figure 12B) and CDS11 (Figure 12C) cells, CyQuant fluorescence reflecting anchorage-independent growth, was significantly modulated by the treatments as demonstrated by one-way ANOVA tests (CS-1: F=6.40, p=0.0013; CDS11: F=8.991, p=0.0002). Post hoc comparisons to vehicle demonstrated significant reductions in colony formation following SMI-L+DOX and SMI-H+DOX treatment of CS-1 cells (Figure 12B), and significant reductions in colony formation associated with all treatments except SMI-L monotherapy in CDS11 cells (Figure 12C).

**Figure 13:**
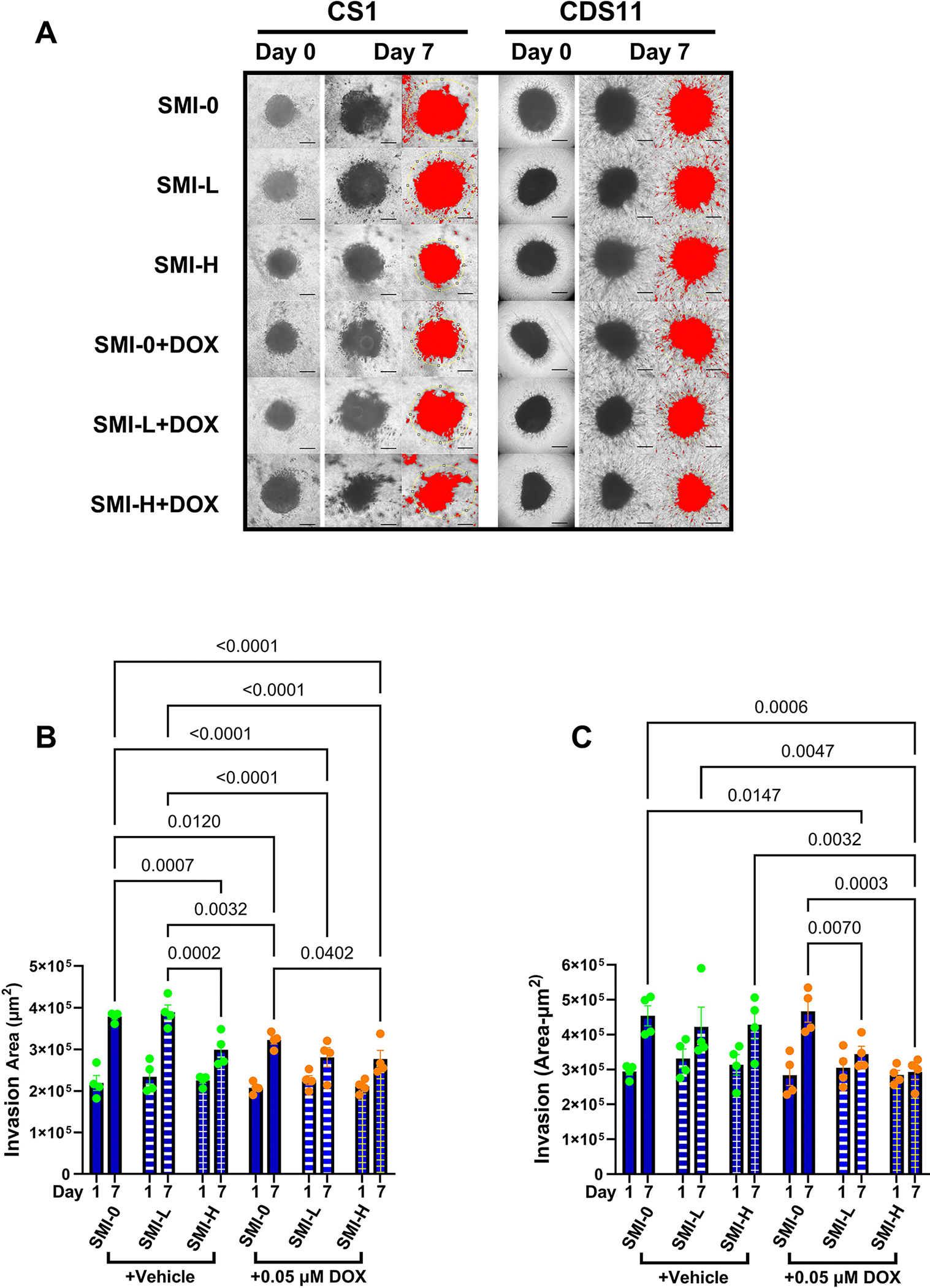
Invasion Assay. A 96-well 3D Spheroid Basement Membrane Extract (BME) Cell Invasion BME assay kit was used to examine the effects of low (3.125 μM) or high (25 μM) SMI-1182, DOX (0.05 μM), or DOX+low- or high-dose SMI1182 on cell invasion relative to vehicle-control. (A) Representative images of Day 1 and Day 7 CS-1 and CDS11 cultures under different treatment conditions. The red pseudo-colored images correspond to the areas measured using Image-J software (U. S. National Institutes of Health). Results of paired Day 1 and Day 7 quantitative analysis of spheroid cross-sectional areas in (B) CS-1 and (C) CDS11 cultures (n=4/treatment group). No significant inter-group differences were detected in Day 1 cultures. Statistical comparisons by ANOVA with post hoc multiple comparisons tests were limited to results obtained on Day 7. Significant p-values are displayed within the panels.

Extracellular matrix invasion in 3D spheroid cultures was measured by image analysis using Image J software (U. S. National Institutes of Health). Example images corresponding to CS-1 and CDS11 cultures are shown in (Figure 13A). The effects of SMI-L, SMI-H, DOX, SMI-L+DOX, and SM-H+DOX were assessed on Days 1 and 7 by two-way ANOVA and post hoc multiple comparisons tests. For CS-1 cells, two-way ANOVA detected significant effects of culture duration (F=143.4; p<0.0001), treatment (F=6.382; p=0.0002) and treatment duration × treatment type interactions (F=4.602, p=0.0024). Post hoc tests demonstrated no significant intergroup differences on Day 1, but significantly reduced invasive growth in cultures treated for 7 days with SMI-H, DOX, SMI-L+DOX, or SMI-H+DOX relative to vehicle and SMI-L (Figure 13B). On the Day 7 endpoint, the mean areas of agar invasion in CS-1 cultures were significantly greater following Vehicle- or SMI-L monotherapy relative to SMI-H, DOX, SMI-L+DOX, and SMI-H+DOX (Figure 13B). In addition, SMI-H+DOX significantly reduced the mean invasive area relative to DOX monotherapy in CS-1 cultures. For CDS11 cultures, two-way ANOVA detected significant effects of culture duration (F=32.17; p<0.0001), treatment (F=2.964; p=0.0243), and treatment duration × treatment type interactions (F=2.47, p=0.05). Post hoc tests demonstrated no significant intergroup differences on Day 1, but significantly reduced agar invasion in SMI-L+DOX and SMI-H+DOX treated cultures relative to vehicle (Figure 13C). In addition, 7-day invasive growth was reduced in SMI-L+DOX relative to DOX, i.e., SMI-0+DOX, and in SMI-H+DOX relative to DOX, SMI-L, and SMI-H.

### Molecular correlates of smi-1182- and dox-mediated inhibition of cs cell survival, motility, and invasive growth

**Figure 14:**
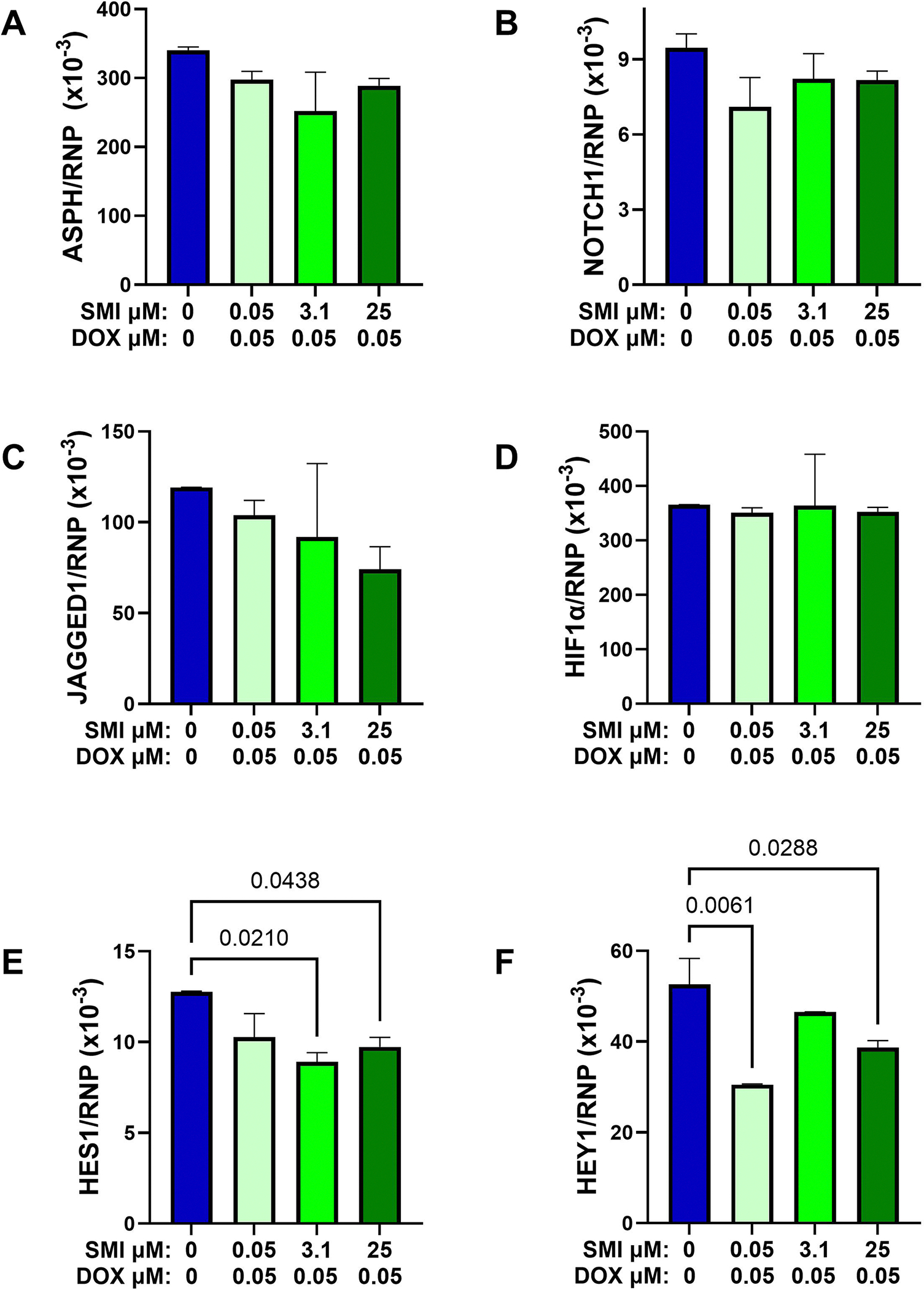
Molecular Pathways of SMI-1182- and DOX-Mediated CS Cytotoxicity Via ASPH and Notch Networks. CS-1 cells were treated for 48 with 0.05 μM, 3.125 μM, or 25 μM of SMI-1182 (SMI) and low-dose (0.05 μM) DOX. (A) ASPH, (B) Notch1, (C) Jagged 1, (D) HIF-1a, (E) HES1, and (F) HEY1 gene expression were measured using a multiplex bead-based RNA hybridization protocol with results normalized to ribonuclear protein (RNP). Graphs depict the mean ± S.D. of results. Inter-group comparisons relative to control were made by ANOVA with post hoc repeated measures test. Significant p-values are shown in the panels.

**Figure 15:**
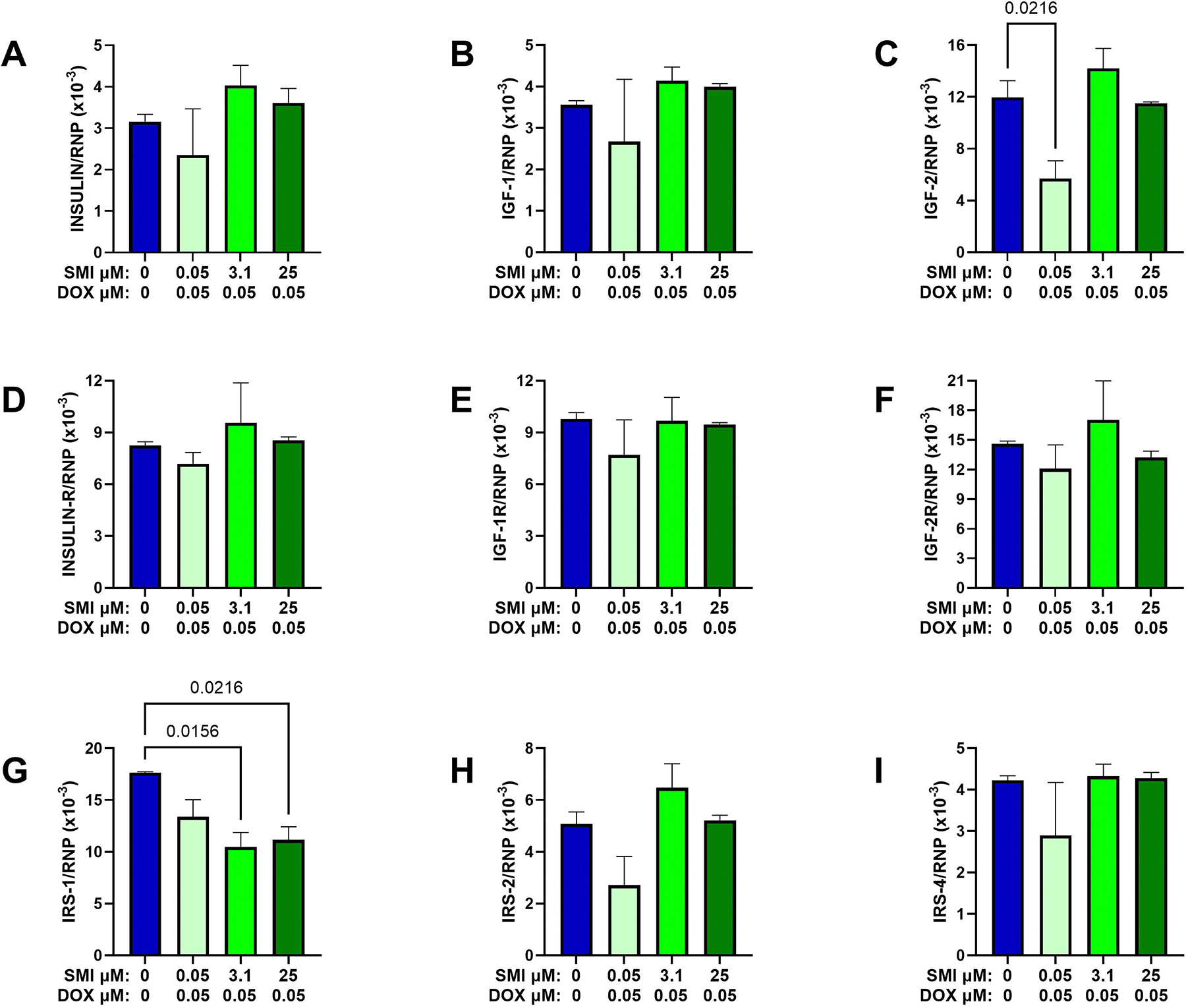
Molecular Pathways of SMI-1182- and DOX-Mediated CS Cytotoxicity Via Insulin, IGF, and IRS Pathways. CS-1 cells were treated for 48 with 0.05 μM, 3.125 μM, or 25 μM of SMI and low-dose (0.05 μM) DOX. (A) Insulin, (B) IGF-1, (C) IGF-2, (D) Insulin-R, (E) IGF-1R, (F) IGF-2R, (G) IRS-1, (H) IRS-2, and (I) IRS-4 mRNA levels were measured using a multiplex bead-based RNA hybridization protocol with results normalized to ribonuclear protein (RNP). Graphs depict the mean ± S.D. of results. Intergroup comparisons relative to control were made by ANOVA with post hoc repeated measures test. Significant p-values are shown in the panels.

Quantigene multiplex RNA hybridization assays were used to examine the effects of SMI-L, SMI-H, and DOX as independent and dual treatments on the expression of ASPH, Notch pathway, and Insulin/IGF upstream networks that mediate cell growth, survival, migration, and invasion. For these studies, we examined the effects of very low (0.05 μM), low (3.125 μM), and high (25 μM) doses of SMI-1182 coupled with 0.05 μM DOX in CS-1 cells. The mRNA levels were normalized to Ribonuclear Protein (RNP). One-way ANOVA tests detected no significant inter-group differences with respect to ASPH, NOTCH1, JAGGED1, or HIF-1α (Figures 14A–14D). In contrast, significant differences were observed for HES1, marked by reduced levels in cells treated with 3.125 μM SMI+DOX or 25 μM SMI+DOX relative to vehicle (Figure 14E), and for HEY1, which was significantly reduced in cultures treated with 0.05 μM SMI+DOX or 25 μM SMI+DOX relative to vehicle (Figure 14F). Regarding the insulin, IGF, and IRS pathways (Figure 15), significant inter-group treatments were detected only for IGF-2 (F=8.965, p=0.03) and IRS-1 (F=6.645, p=0.0494). The post hoc tests demonstrated significantly reduced levels of IGF-2 mRNA in cells treated with 0.05 μM SMI+DOX (Figure 15C) and reduced IRS-1 mRNA in cells treated with 3.125 μM SMI+DOX or 25 μM SMI+DOX relative to vehicle (Figure 15G). Of note is that IGF-2 was more abundantly expressed than insulin or IGF-1 (Figures 15A–15C), IGF-2R was more abundant than insulin-R or IGF-1R (Figures 15D–15-F), and IRS-1 was more abundantly expressed than IRS-2 or IRS-4 (Figures 15G–15-I). The greater abundance of mRNA transcripts supporting IGF-2 and IRS-1 networks in CS-1 cells suggests that significant effects of treatment would likely be mechanistically impactful.

## Discussion

The goals of this study were to characterize the expression of ASPH in human CS cells and examine the benefits of ASPH targeting as a monotherapy, or an enhancer of DOX’s chemotherapeutic effects. In addition, efforts were made to examine the treatment effects on key signaling pathways that modulate ASPH expression and function. ASPH stands out as an important potential therapeutic target in malignancy due to its high levels of expression on cancer cell surfaces, and very low or absent expression in most normal cells [32]. Identifying additional therapeutic targets for CS is a priority set by the American Academy of Orthopaedic Surgeons. To this end, we examined the effects of a small molecule inhibitor of ASPH, SMI-1182, on CS cell viability, cytotoxicity, motility, colony formation, and invasive growth. The outcomes were linked to mechanistic drivers of Notch and insulin/IGF/IRS signaling that are known to impact a broad array of cellular functions linked to malignant tumor behavior.

The study design utilized two established human conventional CS cell lines regarded as histologic Grade 3 (CS-1) or Grade 2 (CDS11). Efforts were made to delineate similarities and differences in ASPH expression and sensitivity to SMI-1182 and DOX. The use of 96-well microculture assays enabled efficient testing of broad dose-range responses with multiple simultaneous replicates under the same experimental conditions. Several complementary assays tested the effects of SMI-1182, in the presence of absence of DOX, on critical functions including cell migration, anchorage-independent growth, colony formation, and invasion, all of which characterize malignant phenotypes.

Although both CS-1 and CDS11 cells exhibited SMI-1182 and DOX dose-dependent reductions in cell viability and increases in cytotoxicity, area-under-curve analysis demonstrated significantly higher IC50’s in CDS11 cells. These findings suggest that cells originating from the lower grade CS were more resistant to SMI-1182 and DOX monotherapy. The dose-response curves corresponding to the effects of dual treatment provided new evidence that the IC50s for DOX-mediated cell loss and cytotoxicity could be significantly lowered for both CS cell lines by the co-administration of SMI-1182. Importantly, although the effectiveness of cell killing over a broad dose range of SMI-1182 was significantly greater at higher compared with lower DOX doses, comparable degrees of CS killing were achieved by dual treatment with SMI+low-dose DOX versus SMI+high-dose DOX or DOX monotherapy. Moreover, composing analysis demonstrated that the SMI-1182+DOX treatment effects were synergistic rather than simply additive. These results suggest that the effectiveness of DOX chemotherapy, even at lower doses, could be substantially enhanced by the co-administration of SMI-1182.

The differential cytopathological effects of SMI-1182 and DOX on CS cells could be due to different mechanisms of cytotoxicity and cell death. Membrane blebbing, i.e, irregular bulging of the plasma membrane, seen with either treatment, likely reflects decoupling of the cytoskeleton from the plasma membrane. Physiologically, the response could occur in the process of cell migration such as in association with cancer cell metastasis [62], or in the process of apoptosis [63]. The prominent cell shrinkage with nuclear condensation associated with DOX, with or without SMI-1182 is consistent with DOX’s known mechanisms of cancer cell killing, including necrosis, cytotoxicity, autophagy, and apoptosis [64]. In contrast, the cell swelling, pallor, nuclear loss and fragmentation noted more in SMI-1182 treated cells likely represent apoptosis as a prominent mechanism of cell death, corresponding with known effects of Notch pathway inhibition [65–67].

Previous studies showed that ASPH has functional roles in mediating cell motility and invasion via its catalytic activity that hydroxylates Asp and Asn residues in the EGF-like domains of Notch and Jagged [32]. SMI-1182 was shown to reduce cell motility by inhibiting expression of Notch transcription factors rather than ASPH, Notch, or Jagged [34]. Herein, similar observations were made in CS cells treated with SMI-1182 monotherapy. However, dual treatment with SMI-1182 and DOX also reduced ASPH immunoreactivity but not mRNA. Furthermore, SMI-1182 did not reduce FB50 immunoreactivity which targets Humbug, a truncated version of ASPH that lacks the C-terminal catalytic domain utilized for Notch activation and critical for promoting cell motility [68]. The absence of FB50-ASPH suppression by SMI-1182 reinforces specificity of the SMI for ASPH’s catalytic domain. These findings suggest that the inhibition of ASPH’s catalytic activity coupled with reduced ASPH protein expression account for the synergistic responses to the dual SMI-1182+DOX treatments. The additional finding that SMI-1182+DOX inhibited IRS-1 expression is significant because reduced signaling through insulin/IGF/IRS pathways leads to increased glycogen synthase kinase 3β (GSK-3β) activity, which reduces ASPH protein expression via increased degradation, without altering its mRNA [61,69], as observed herein.

The functional assays revealed that CS-1 and CDS11 cells had non-identical responses to DOX, SMI-1182, and combined treatments. Under all experimental conditions, the mean percentages of motile cells were significantly higher in CS-1 than CDS11 cultures, corresponding with the higher histologic grade of the original tumor. Importantly, CDS11 but not CS-1 cells exhibited significant reductions in cell motility following dual versus mono-therapy. Colony formations were larger with CDS11 cells, and although dual SMI-1182+DOX treatments significantly reduced colony formation in both CS-1 and CDS11 cultures, the proportional reductions were larger for CS-1 cells. In contrast, the invasion assay results were similar for CS-1 and CDS11 cells in terms of the inhibitory effects of dual treatments on invasive growth measured on the Day 7 assay endpoint. In essence, the combined SMI-1182+DOX treatments were more effective for inhibiting cellular functions utilized with invasion and metastatic spread of malignant neoplastic cells, but the responses were not identical for CS-1 and CDS11 cells. Additional studies are needed to understand predictors of optimum therapeutic responses. Importantly, co-administration of SMI-1182 appears to render DOX more cytotoxic, and therefore, SMI-1182 could be used to enhance the therapeutic responses to DOX or lower the dose of DOX to minimize its off-target and toxic effects.

Further studies examined signaling pathway mRNA transcripts linked to ASPH expression and Notch to better understand the mechanistic responses to monotherapy versus combination therapy. The main findings were that dual treatment with SMI-1182+DOX significantly reduced HES1, HEY1, and IRS-1. In addition, at the lowest SMI + DOX combination dose, IGF-2 mRNA was significantly reduced relative to vehicle. The reductions in HES1 and HEY1 mRNA expression correspond with SMI-1182’s inhibitory effects on ASPH activation of Notch and the attendant expression of critical transcription factors [28,29,36]. Similar responses were observed previously in experiments designed to study the effects of ASPH inhibition via mutagenesis or silencing (siRNA or lentivirus) [34, 70–72]. The finding of reduced IRS-1 expression linked to SMI-1182+DOX treatments is novel and likely important in relation to the transmission of signals utilized for cell growth, survival, and motility functions [27]. Mechanistically, IGF transmits signals through IRS-1, driving a broad range of responses that include increases in ASPH expression at both mRNA and protein levels [27]. Therefore, the SMI-1182+DOX mediated significant reductions in A85G6-ASPH (full-length protein including the catalytic domain) immunoreactivity could be explained by the inhibition of IRS-1. The combined inhibition of IRS-1 expression with ASPH’s catalytic activity likely accounts for the significant reductions in chondrosarcoma cell motility, colony formation, and invasive growth. The aggregate findings support the concept that SMI-1182+DOX dual therapy could provide more effective treatment compared with the current standard DOX monochemotherapeutic regimen for recurrent chondrosarcoma in humans.

## Conclusions

- This study successfully characterized ASPH expression in human CS cells and demonstrated ASPH’s role in CS cell motility and invasion via targeting with the small molecule inhibitor, SMI-1182.
- SMI-1182 and DOX independently inhibited CS cell viability and increased cytotoxicity in dose-dependent manners.
- Combination treatments with SMI-1182 and DOX had synergistic cytotoxic effects compared to monotherapy. The adjuvant therapeutic effects of SMI-1182 were evidenced by the lower DOX doses needed to kill CS cells.
- Treatment with SMI-1182 + DOX more effectively inhibited motility and invasive CS cell growth compared with either monotherapy.
- Mechanistically, SMI-1182+DOX inhibited Notch and IRS signaling mechanisms, which are highly active in aggressive malignant neoplasms, including CS.
- SMI-1182 inhibition of ASPH-driven Notch pathway activation is a promising mechanistic strategy for treating CS in humans. Combination therapy with DOX plus SMI-1182 could improve clinical outcomes from CS compared with DOX monotherapy.

## Supplementary Material

Supplementary Figure 1

raw western images

## Figures and Tables

**Figure 16:**
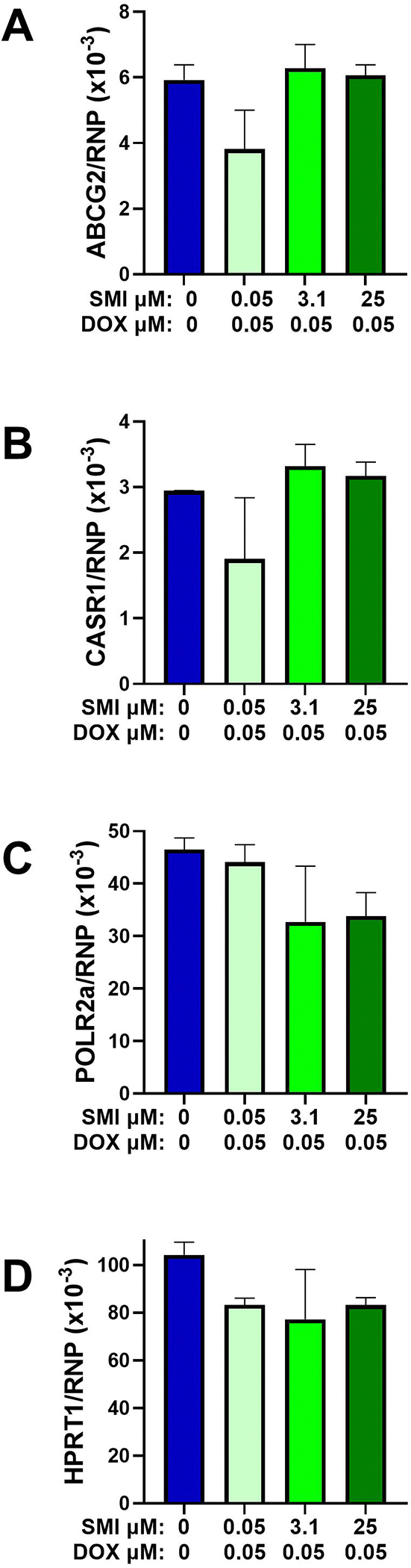


**Table 1:** Antibody Sources, Characteristics and Concentrations Used

Antibody	Source	Type	Concentration Used
FB-50 (ASPH)*	Liver Research Center, Brown University Health	Mouse monoclonal	0.845 μg/mL
A85G6 (ASPH)*	Liver Research Center, Brown University Health	Mouse monoclonal	1.3 μg/mL
Vimentin	RV-202; Abcam ab8978	Mouse monoclonal	2.5 μg/mL
RPLPO*	Santa Cruz; sc-293260	Mouse monoclonal	1 μg/mL
PCNA*	BD Bioscience; #610664	Mouse monoclonal	0.5 μg/mL
β-Actin	Santa Cruz (C4); sc-47778	Mouse monoclonal	0.2 μg/mL
HRP-conjugated secondary*	Thermo Fisher Scientific; Product Number: 31430 (anti-mouse); 31461 (anti-rabbit)	Anti-mouse + anti-rabbit	0.04 μg/mL

* ASPH=aspartyl-asparaginyl-β-hydroxylase; RPLPO=Large Acidic Ribonuclear Protein; PCNA=proliferating cell nuclear antigen; HRP=horseradish peroxidase

**Table 2:** Two-way ANOVA Table for DOX Dose-Dependent Shifts in MTT, Viability, and Cytotoxicity

Analysis	CS Grade Factor: F-Ratio	p-value	DOX Dose Factor: F-Ratio	p-value	CS Grade × DOX Dose: F-Ratio	p-value
MTT	6.794	<0.0155	282.6	<0.0001	42.01	<0.0001
H33342	0.002	N.S.	221.4	<0.0001	8.469	0.0005
G6PD	0.791	N.S.	492.0	<0.0001	30.04	<0.0001

CS1 and CDS11 cells were treated with a dose range of SMI-1182 + 0, 0.05μM, 0.5μM or 1μM DOX for 48h. MTT activity, H33342 fluorescence, and G6PD release were measured in CS1 and CDS11 cells. The results of two-way ANOVA tests are shown. For all analyses, (DFn, DFd)=1,24 for CS Grade factor, and (DFn, DFd)=3,24 for DOX dose factor and CS Grade × DOX dose interactions. See Supplementary Figure 1.

**Table 3:** Two-way ANOVA Table for CS Motility and Invasion ATP-Lite Assays

Analysis	Treatment Factor: F-Ratio	p-value	Motility Factor: F-Ratio	p-value	Treatment × Motility: F-Ratio	p-value
CS1 Motility	0.513	N.S.	110.4	<0.0001	4.474	0.0003
CS1 Motility/Invasion	0.775	N.S.	109.2	<0.0001	3.732	0.0013
CDS11 Motility/Invasion	3.551	0.021	154.6	<0.0001	13.08	<0.0001

CS1 and CDS11 cells were treated with Vehicle (control), SMI-1182, DOX, or SMI-1182+DOX. Directional motility and invasion were measured in Boyden chamber-type assays in which the membranes dividing upper and lower chambers were uncoated to measure directional motility in CS1 cells or were coated with Geltrex to measure motility and invasion in CS1 and CDS11 cells. The results of two-way ANOVA tests are shown. For all analyses, (DFn, DFd)=3,48 for treatment and motility factors, and (DFn, DFd)=9,48 for treatment × motility interactions.
