# Supplementary material for: Chondrosarcoma: Adjuvant Therapeutic Effects of the ASPH Small Molecule Inhibitor, SMI-1182, With Doxorubicin
Source: Jpn J Cancer Oncol Res. Author manuscript; Available in PMC 2026 Jul 18. (PMC13378441)
Supplement: Supplementary Figure 1 [file NIHMS2162193-supplement-Supplementary_Figure_1.docx]

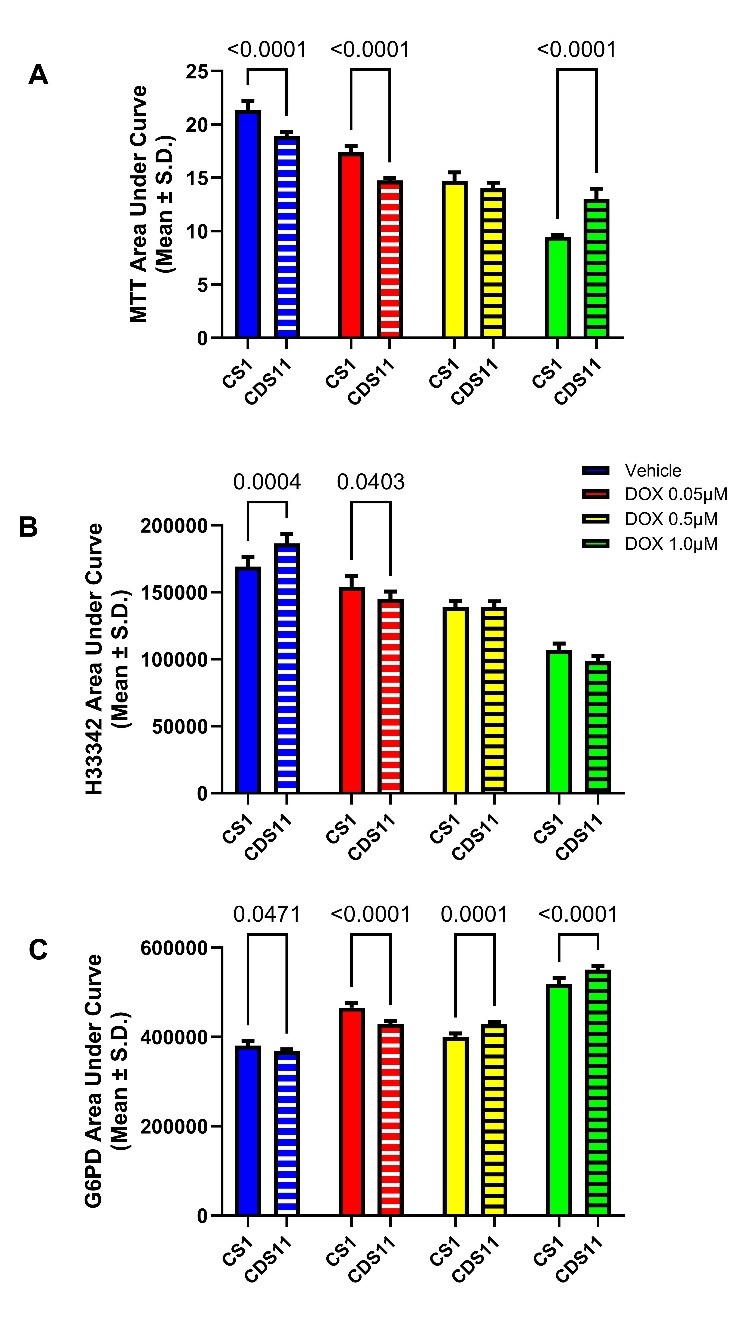


Supplementary Figure S1: Comparisons of CS1 and CDS11 cellular responses to a dose-range of treatment with SMI-1182 plus 0, 0.05, 0.5 or 1 µM DOX. Graphs depict the mean + S.D. of area under curve (AUC) analysis of (A) MTT activity, (B) Hoechst H33342 fluorescence (viability), and (C) G6PD release (cytotoxicity). The Two-way ANOVA results are shown in Table 2. Significant post hoc results are shown over the graphs.
