# Supplementary material for: Chondrosarcoma: Adjuvant Therapeutic Effects of the ASPH Small Molecule Inhibitor, SMI-1182, With Doxorubicin
Source: Jpn J Cancer Oncol Res. Author manuscript; Available in PMC 2026 Jul 18. (PMC13378441)

Molecular marker (WB)

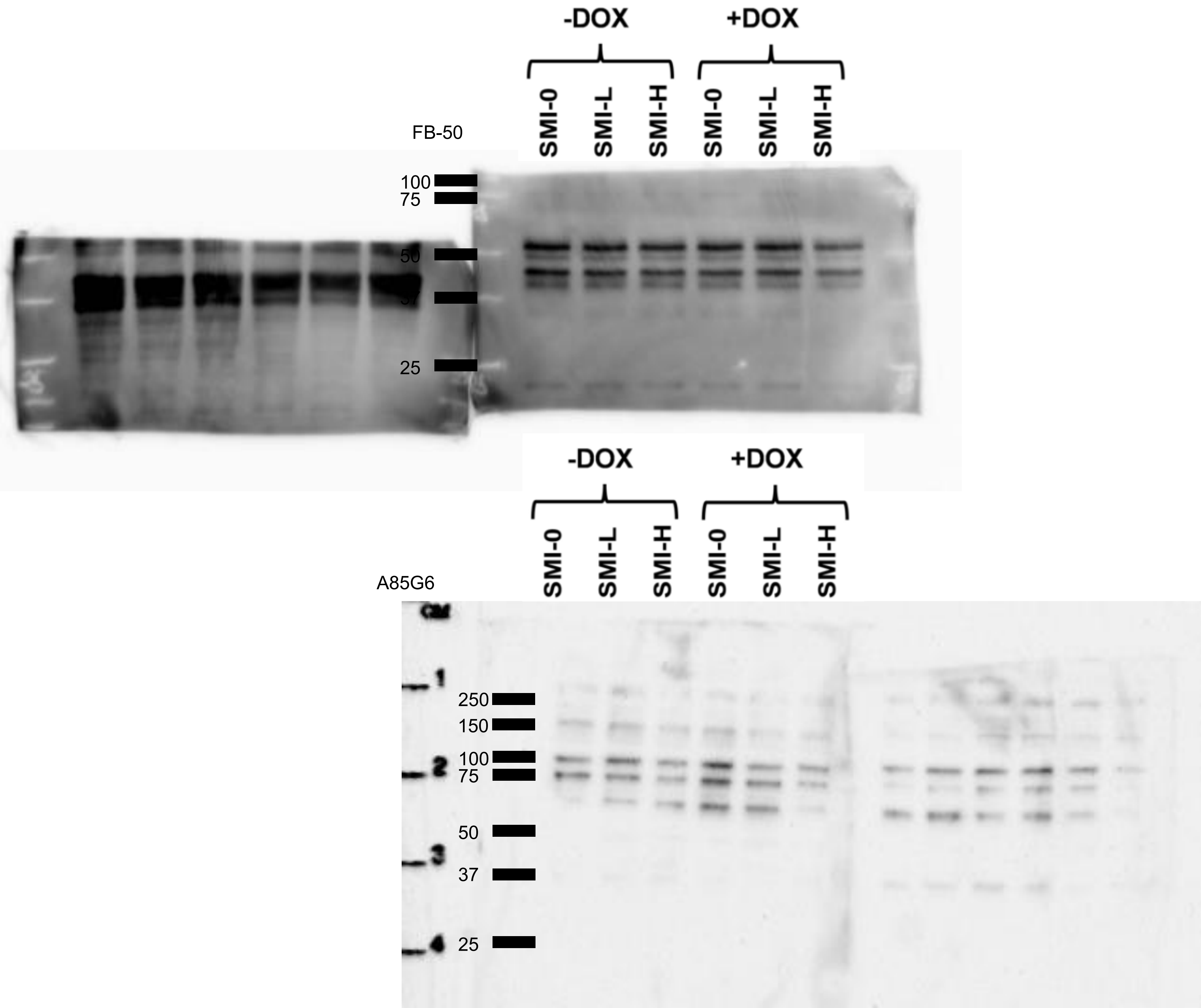

Molecular marker (WB)

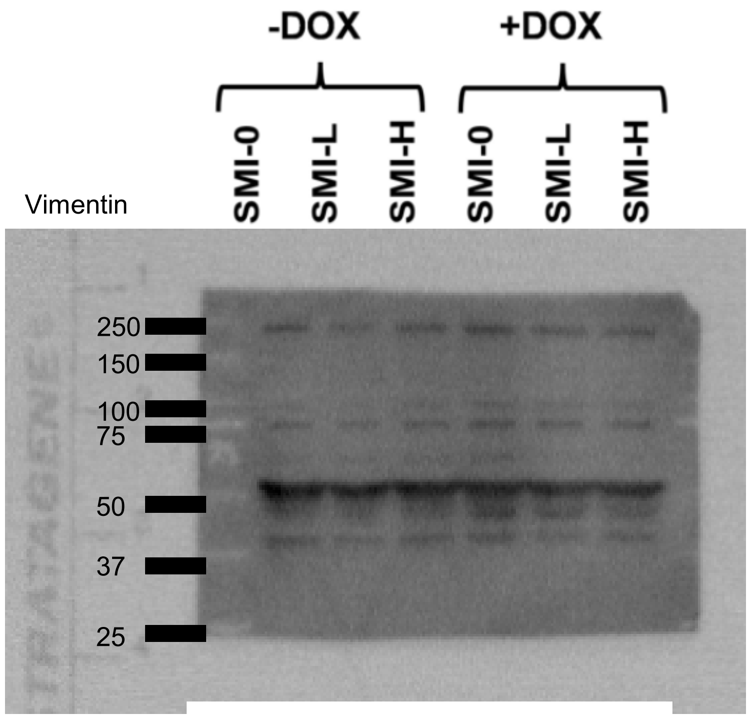

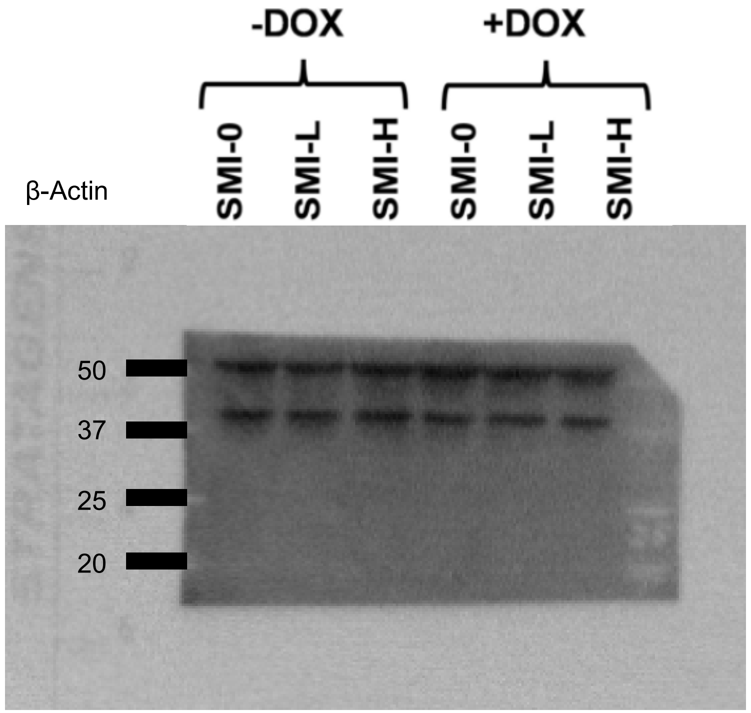

Densitometry (ImageJ)

FB-50

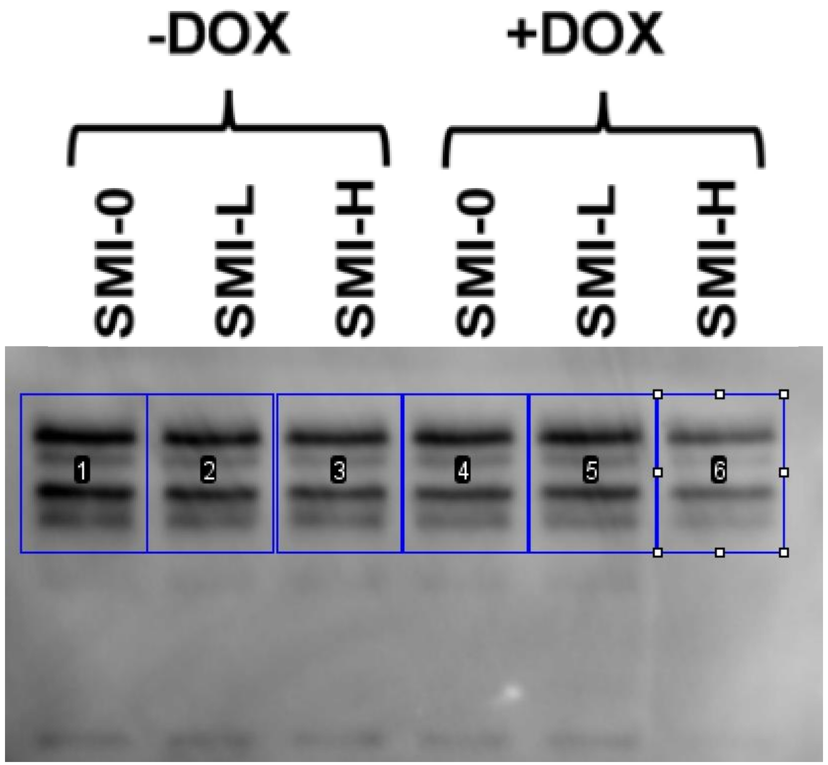

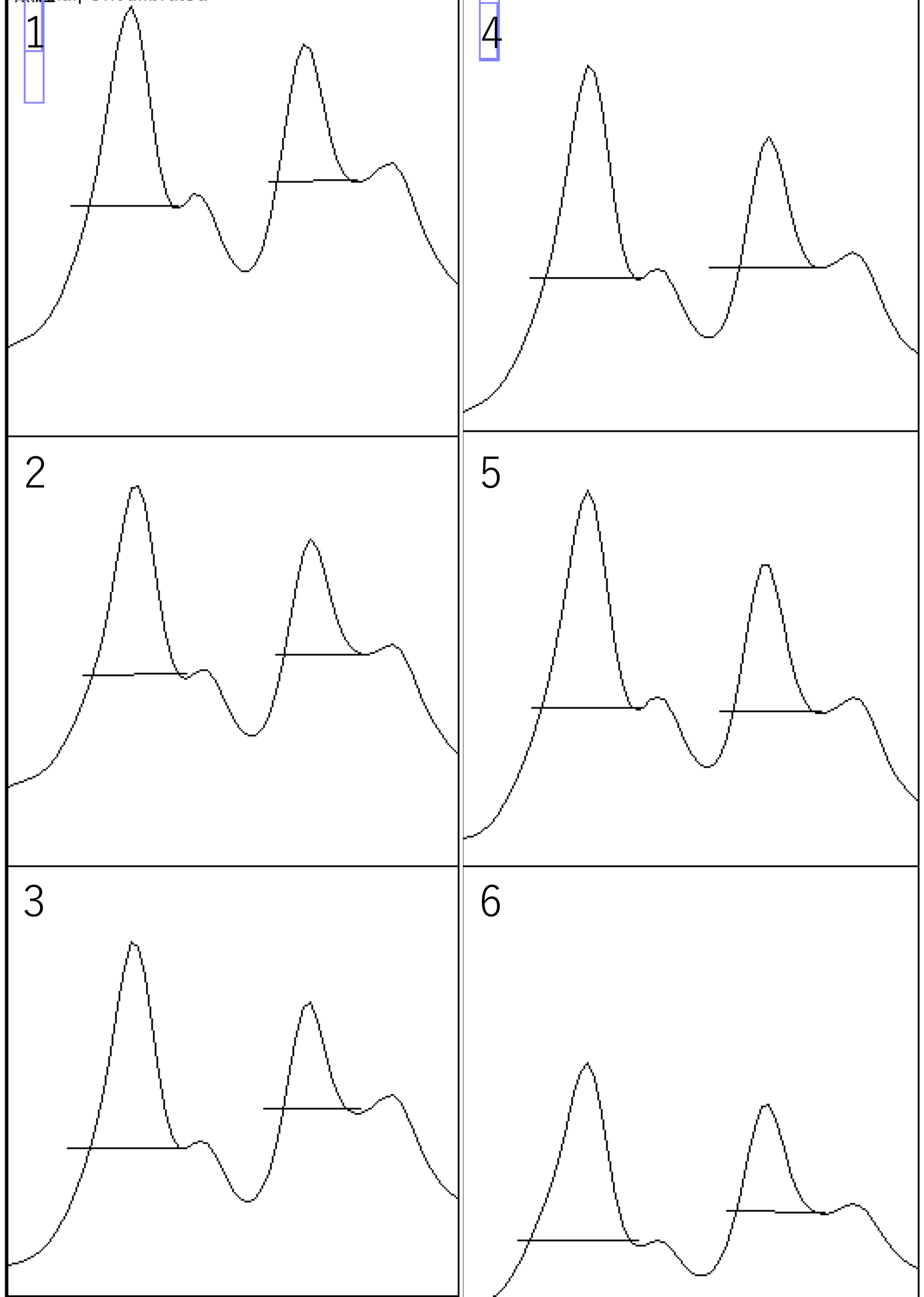

Number	Sample	Intensity	Ratio (β-Actin)
1	SMI-0 DOX-	5465.958	2.071095
2	SMI-L DOX-	5016.19	2.085466
3	SMI-H DOX-	5082.751	1.884191
4	SMI-0 DOX+	4978.569	2.64609
5	SMI-L DOX+	4971.069	2.459092
6	SMI-H DOX+	3264.483	1.853978

Densitometry (ImageJ)

A85G6

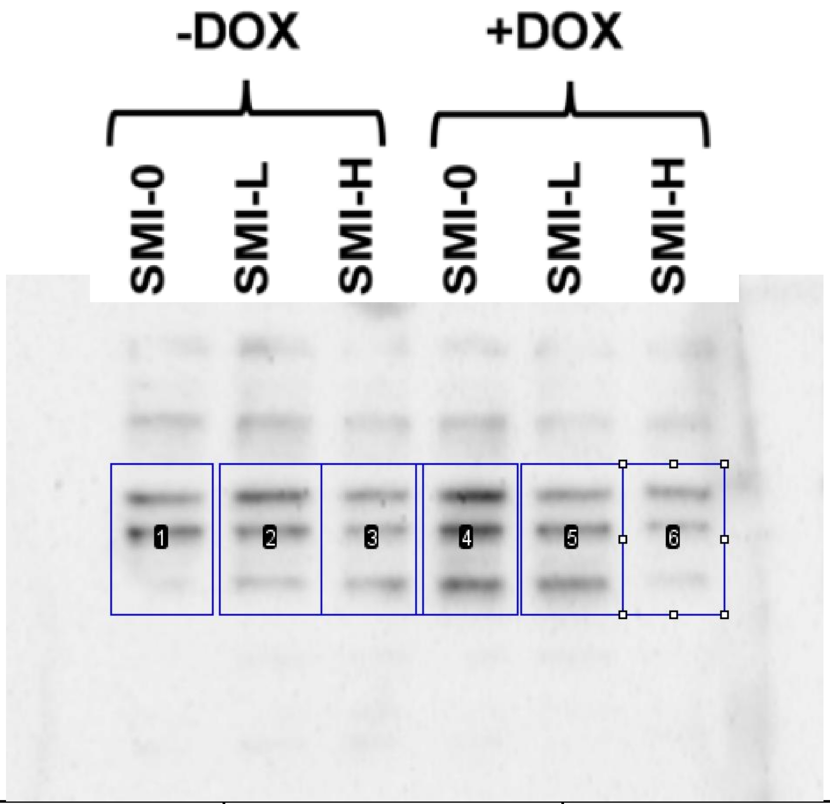

Number	Sample	Intensity	Ratio (β-Actin)
1	SMI-0 DOX-	4219.645	1.598857
2	SMI-L DOX-	4438.979	1.845492
3	SMI-H DOX-	2458.703	0.911449
4	SMI-0 DOX+	5443.645	2.893277
5	SMI-L DOX+	3182.15	1.574148
6	SMI-H DOX+	2180.507	1.238362

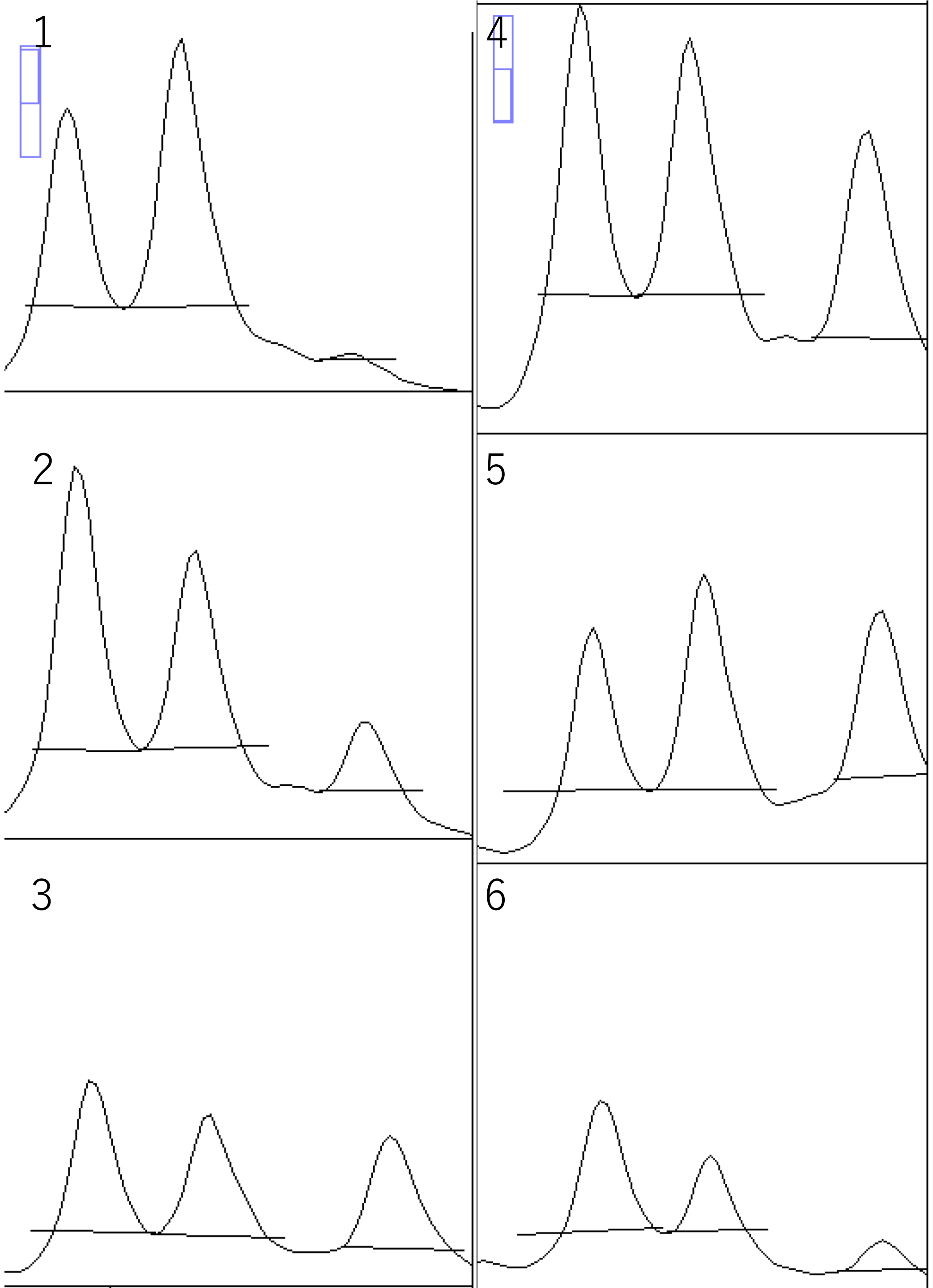

Densitometry (ImageJ)

Vimentin

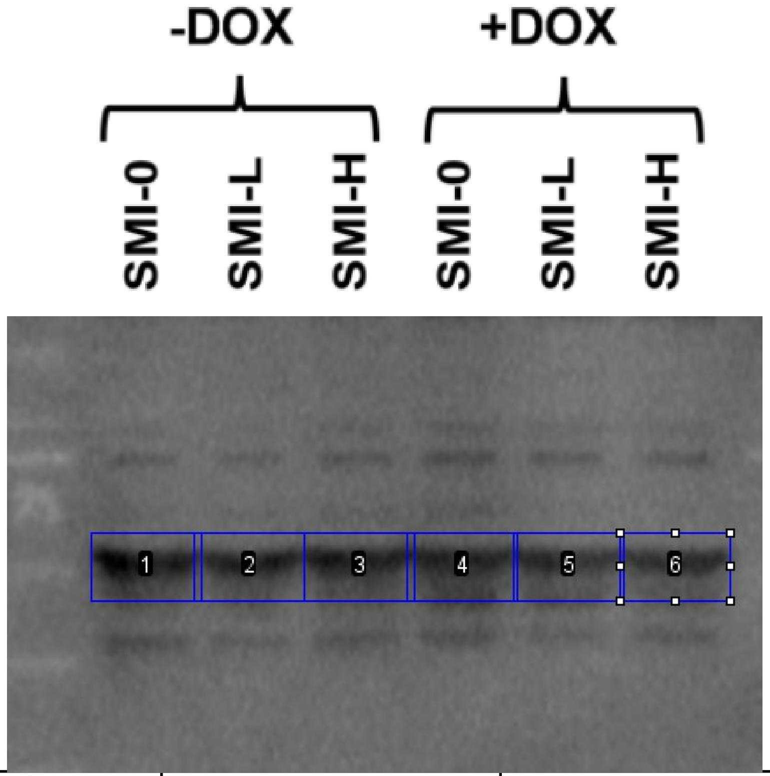

Number	Sample	Intensity	Ratio (β-Actin)
1	SMI-0 DOX-	2650.517	1.004302
2	SMI-L DOX-	2572.87	1.069663
3	SMI-H DOX-	2585.284	0.958373
4	SMI-0 DOX+	1768.456	0.939928
5	SMI-L DOX+	1863.689	0.921931
6	SMI-H DOX+	1928.481	1.095231

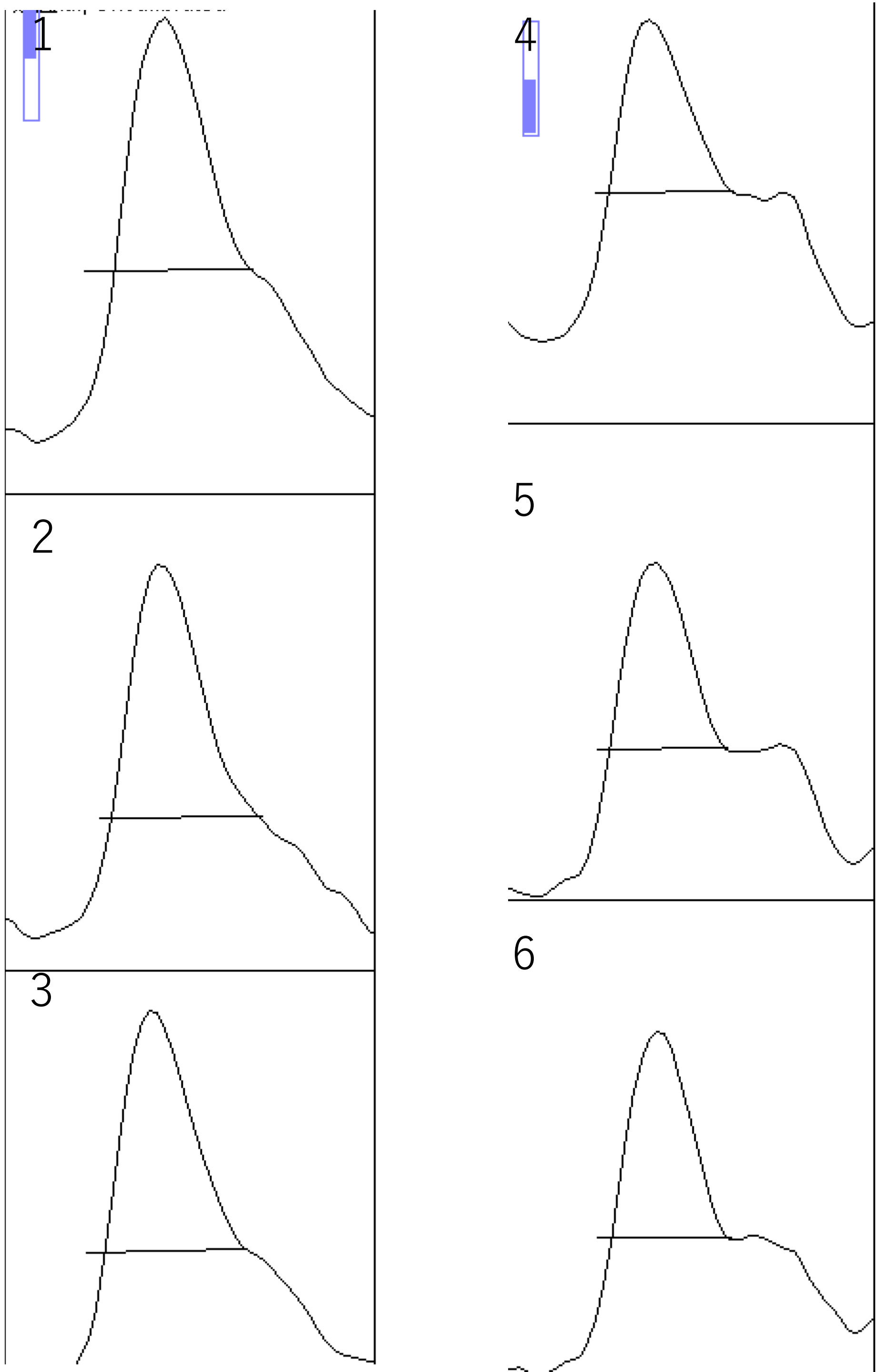

Densitometry (ImageJ)

β-Actin

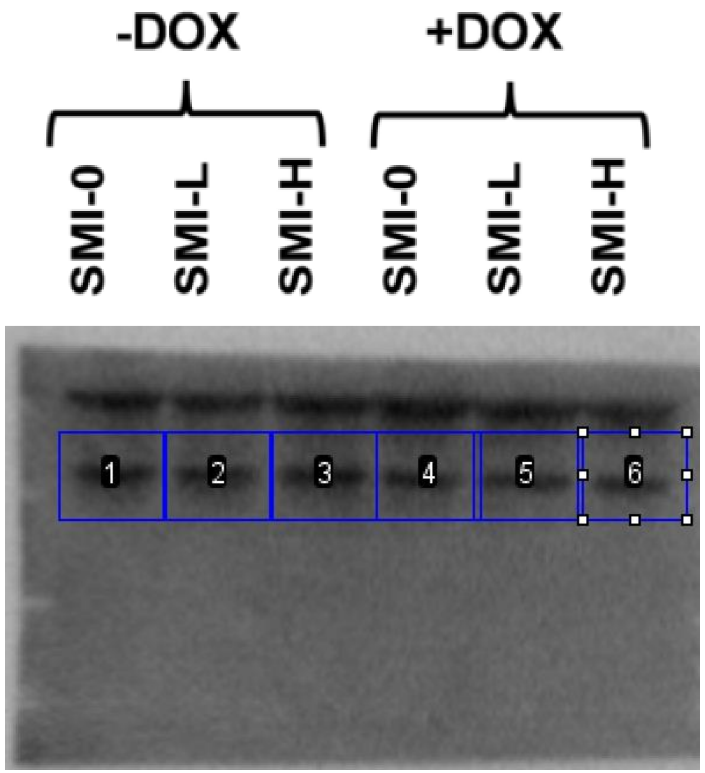

Number	Sample	Intensity	Ratio (β-Actin)
1	SMI-0 DOX-	2639.163	
2	SMI-L DOX-	2405.31	
3	SMI-H DOX-	2697.577	
4	SMI-0 DOX+	1881.481	
5	SMI-L DOX+	2021.506	
6	SMI-H DOX+	1760.799	

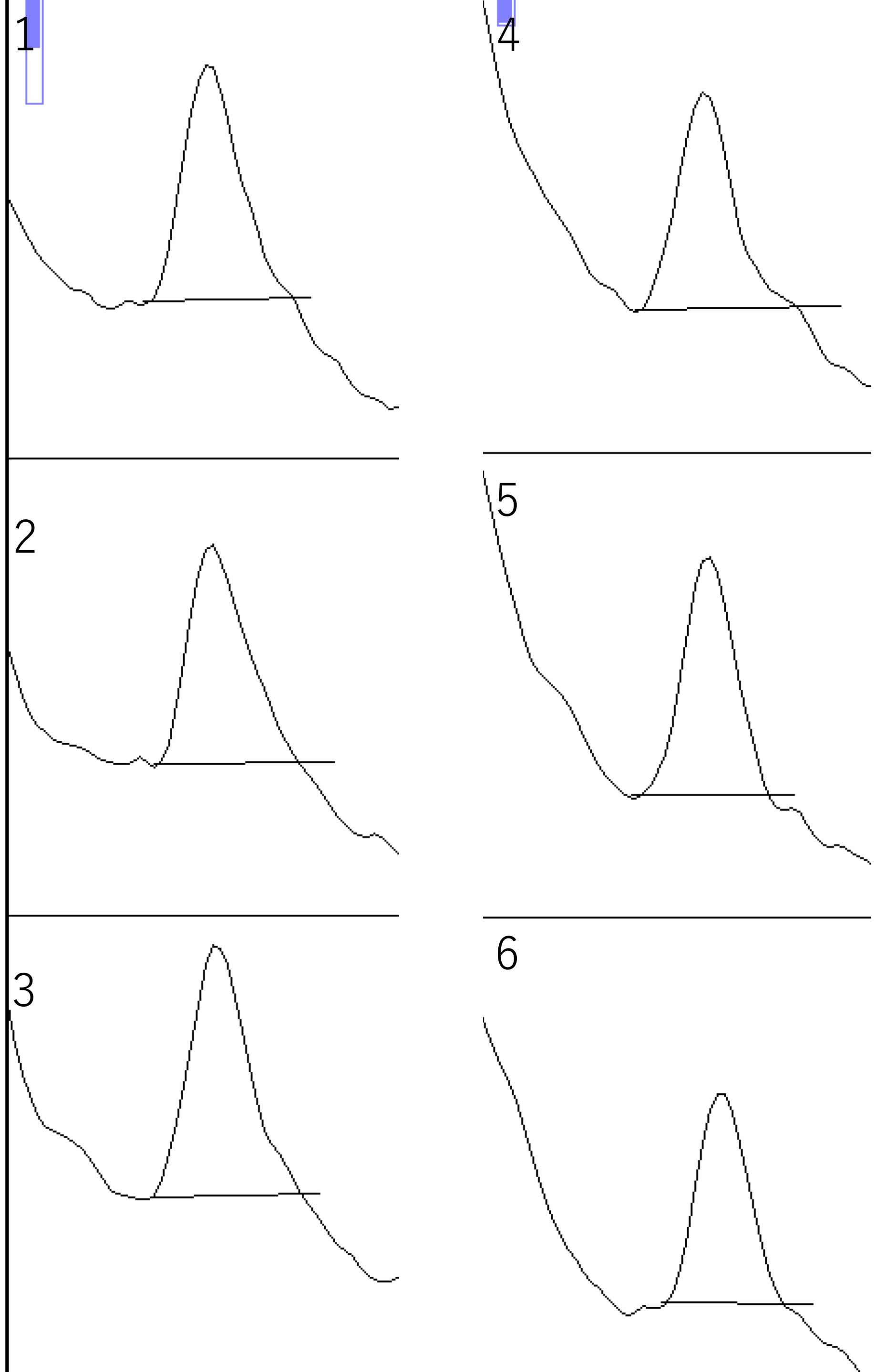

Supplement: raw western images [file NIHMS2162193-supplement-raw_western_images.pdf]
